# TiO_2_-SiO_2_ Coatings with a Low Content of AuNPs for Producing Self-Cleaning Building Materials

**DOI:** 10.3390/nano8030177

**Published:** 2018-03-20

**Authors:** Manuel Luna, Juan J. Delgado, M. L. Almoraima Gil, María J. Mosquera

**Affiliations:** 1 TEP-243 Nanomaterials Group, Department of Physical-Chemistry, Faculty of Sciences, University of Cadiz, 11510 Puerto Real, Spain; manuel.luna@uca.es (M.L.); almoraima.gil@uca.es (M.L.A.G.); 2 Department of Inorganic Chemistry, CASEM, University of Cadiz, 11510 Puerto Real, Spain; juanjose.delgado@uca.es

**Keywords:** photocatalyst, self-cleaning, building, Au-TiO_2_/SiO_2_

## Abstract

The high pollution levels in our cities are producing a significant increase of dust on buildings. An application of photoactive coatings on building materials can produce buildings with self-cleaning surfaces. In this study, we have developed a simple sol-gel route for producing Au-TiO_2_/SiO_2_ photocatalysts with application on buildings. The gold nanoparticles (AuNPs) improved the TiO_2_ photoactivity under solar radiation because they promoted absorption in the visible range. We varied the content of AuNPs in the sols under study, in order to investigate their effect on self-cleaning properties. The sols obtained were sprayed on a common building stone, producing coatings which adhere firmly to the stone and preserve their aesthetic qualities. We studied the decolourization efficiency of the photocatalysts under study against methylene blue and against soot (a real staining agent for buildings). Finally, we established that the coating with an intermediate Au content presented the best self-cleaning performance, due to the role played by its structure and texture on its photoactivity.

## 1. Introduction

Today, the concentration of pollutants is significantly high in big cities and industrial environments. Atmospheric aerosol pollutants produce visible stains on buildings. Specifically, small particles and greasy deposits are adhered to building surfaces by organic binders such as hydrocarbons and fatty acids [1]. These pollutants promote a significant change in the aesthetic of historic and modern buildings of our cities and, consequently, it is responsible for costs investments in building conservation. 

The use of photoactive building materials provides a possible solution, because organic soiling deposited on their surfaces can be decomposed to water and CO_2_ by the action of sun light alone [2]. Since TiO_2_ photoactivity was discovered [3], it has become the most popular photocatalyst for several reasons: stability, availability, low cost, lack of toxicity and excellent photocatalytic properties [4]. Regarding the field of construction, TiO_2_ has been traditionally employed as a white pigment and as a photocatalyst from 1990 [2]. Photoactive surfaces have been produced on a great variety of building materials, such as glass [5], ceramics [6], roof tiles [7] and especially in stones [8,9,10,11,12,13,14], by applying, mainly, TiO_2_ nanoparticles (TiO_2_NPs) dispersed in a solvent (water or volatile organic compunds (VOCs)), as a coating. It is demonstrated that effective self-cleaning and even depolluting surfaces are achieved. However, not enough attention has been paid to the durability of these coatings, a crucial property because they are exposed to outdoor conditions. As reported in the literature, TiO_2_NPs are not suitably adhered to building materials [15,16,17,18]. In addition, cracks resulting from the accumulation of TiO_2_NPs are commonly produced [11,18,19].

Recently, we have designed long-lasting photoactive coatings for building stones by adding TiO_2_NPs into a SiO_2_ precursor sol in the presence of n-octylamine [16,20,21]. The low-viscosity sol can penetrate into the pore structure of substrates and its in situ gelling produces a crack-free TiO_2_/SiO_2_ nanocomposite [16], which is firmly adhered to substrates. The use of siloxanes to immobilize TiO_2_ has also been explored by other researchers in order to prepare durable and well-adhered photoactive coatings on concretes [15,22].

Another drawback of TiO_2_ for application on building materials can be associated to its absorption, being exclusively localized in the ultraviolet range, which only constitutes 3–5% of solar light. Therefore, enhancing the TiO_2_ photoactivity is an important challenge and, moreover, extends its absorption into the visible range, as 45% of solar light is constituted by visible light. Several methods to increase the TiO_2_ photoactivity have been described, by using noble metals [23], metal cations [24], non-metal [25] or organic compounds [26]. The use of nanostructured noble metals is one of the most popular choices for improving TiO_2_ photoactivity, with silver nanoparticles (AgNPs) and gold nanoparticles (AuNPs) being the most widely used [27,28,29,30,31,32]. The metal nanoparticles in contact with TiO_2_ act as an electron reservoir, reducing the recombination of electron-hole pairs [33]. They also have a high localised surface plasmon resonance (LSPR), the light absorption of which produces effects, such as, generation of electron-hole pairs, local heating, or increasing the electric field around, which can promote the TiO_2_ photoactivity [34]. In the specific case of buildings, Ag has been added to TiO_2_ to increase the photoactivity of coatings [35,36,37]. Recently, we have also included a silver precursor to increase the activity of TiO_2_-SiO_2_ photocatalysts [38].

The use of AuNPs instead of AgNPs, in order to improve TiO_2_ photoactivity in coatings applied on buildings, can present significant advantages: (1) The maximum absorption of LSPR of AgNPs is localized around 400 nm, near to UV light, whereas the LSPR of AuNPs is between 500 and 600 nm, the range where solar light is more intense [39]; (2) AgNPs are highly reactive and they can be oxidized in the presence of O_2_, as in outdoor conditions, and this oxidation is promoted as AgNPs are in contact with TiO_2_ with its higher reduction potential [32], whereas AuNPs are considered inert [40]; and, (3) AuNPs are excellent catalysts alone [41] and consequently they can improve other interesting effects, such as the removal of CO and organic compounds by oxidation.

However, it is important to control the amount of AuNPs in coatings because an excess of gold on a TiO_2_ surface can promote some negative effects: (1) a recombination of photo-induced electron-hole pairs [42,43]; (2) a preferential light absorption by gold rather than TiO_2_ [44,45]. In both cases, these drawbacks were observed for Au/TiO_2_ in a proportion above 2 wt %.

As far as we know, the application of Au-TiO_2_ photocatalysts on building materials has scarcely been investigated. Specifically, Bergamonti et al. [46] prepared a sol containing Au-TiO_2_ NPs and it was applied on building stone by brushing. The coatings produced on the stone presented significant cracks related to aggregation of NPs. Regarding photoactivity, no increase was observed due to the addition of gold.

The objective of the present work is to develop effective and long-term Au-TiO_2_/SiO_2_ coatings for building materials. Au and TiO_2_ NPs were incorporated into a starting sol containing silica oligomer and n-octylamine. The integration of Au and TiO_2_ NPs into a silica matrix produces a well-adhered and continuous coating on the building material surface, promoting a high durability [21]. The surfactant plays several roles: (1) to catalyse sol-gel transition of the silica precursor [47,48], and, (2) to create a mesoporous silica gel network that prevents cracking [47,48] and that provides the access of contaminants to photoactive centres, improving photoactivity [21,49]. The Au content was modified in the study in order to investigate its influence in photoactivity. Since a gold excess produces negative effects [43,45] and increases the photocatalyst price, low Au contents (below 0.25 wt %) were employed in the study.

A complete characterization of structure, texture and optical properties of the photocatalysts was carried out and they were applied on a typical building stone in order to evaluate their properties as self-cleaning coatings. Self-cleaning activity was evaluated by studying the decolourization of solid dyes deposited on the coated stones. According to the literature [18,50,51], the changes in the coordinates of a color space is the method most used to evaluate the self-cleaning properties of building materials. This is a simple and rapid strategy but it presents a clear subjectivity, because the measure is associated with visual perception [52,53]. In addition, this methodology can promote a misinterpretation because it does not characterize dye degradation. In this work, we compare the color space method and the application of Kubelka-Munk theory, that describes light absorption in a thin layer of dye deposited on a non or low absorbent substrate [54].

## 2. Materials and Methods

### 2.1. Synthesis of AuNPs

These were prepared according to a green synthesis route [55] based on a synthesis previously developed by our group [56,57]. This synthesis was selected because it is simple and the AuNPs obtained have a wide LSPR band. Drago leaves (Dracaena Draco) were employed as the reducing and stabilizing agent. Specifically, an aqueous extract of Drago leaves (5% *w*/*v*) was prepared under ultrasonic agitation at 50% amplitude, using a Sonopuls HD2200 ultrasonic homogenizer from Bandelin (Berlin, Germany), for 10 min. Next, the extract was filtered.

**Figure 1:**
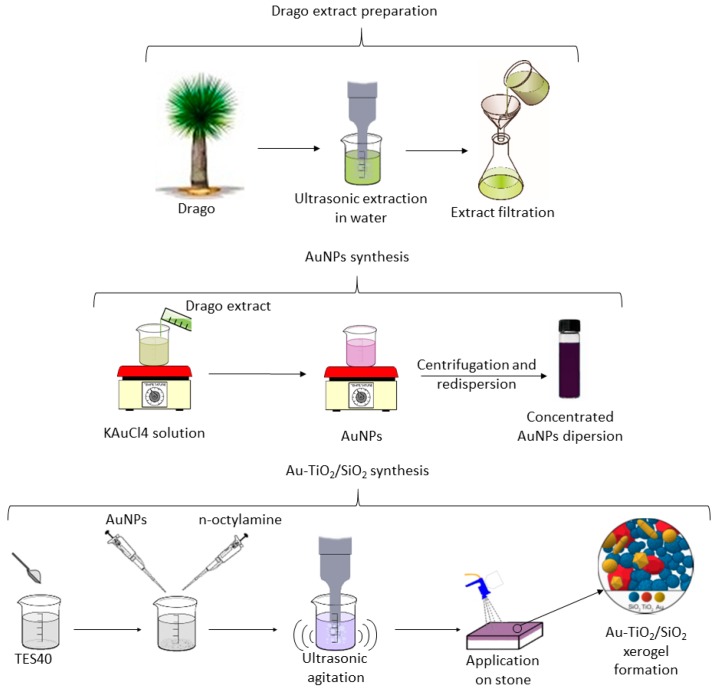
Au-TiO_2_/SiO_2_ route synthesis and application on the stone samples.

KAuCl_4_ was employed as Au precursor. An aqueous 0.47 mM KAuCl_4_ (Sigma-Aldrich, St. Louis, MO, USA) solution was heated to 60 °C and then the Drago extract was added, with a 4:1 solution/extract ratio (*v*/*v*). After 15 min, the dispersion becomes purple, confirming the presence of AuNPs. After 24 h, the Au dispersion was centrifuged at 3000 revolutions per minute (RPM) for 3 min in order to remove the extract residues. The samples were characterized using a JEOL 2010F TEM/STEM microscope (Musahino, Japan) operated at 200 kV. This equipment has a spatial resolution of 0.19 nm in High Resolution Transmission Electron Microscopy (HRTEM) mode. High Angle Annular Dark Field Scanning Transmission Electron Microscopy (HAADF-STEM) images were recorded using an electron probe of 0.5 nm and a camera length of 10 cm. The AuNPs size was determined by measuring more than 300 particles from several HAADF-STEM images at the same magnification. Finally, the AuNPs were precipitated by centrifugation at 13,000 relative centrifugal force (RFC) and re-dispersed in water (Au concentration: 1.5 mg/mL). A scheme of the process is included in Figure 1.

### 2.2. Au-TiO_2_/SiO_2_ Synthesis

The following reagents were employed: a silica oligomer, TES40 WN (Wacker, Munich, Germany), an ethoxysilane (average degree of polymerization of 5) providing approximately 41% of silica upon complete hydrolysis, n-octylamine (Sigma-Aldrich, St. Louis, MO, USA), and, commercial TiO_2_ particles VP Aeroperl P25/20 (Evonik, Essen, Germany) consisting of a granulated version of P25 TiO_2_ particles, with an average particle size of 20 μm, a primary particle size of 21 nm and a surface area of 50 ± 15 m^2^. In the synthetic procedure, silica oligomer was mixed with n-octylamine, TiO_2_NPs and AuNPs dispersion previously prepared were mixed, under high-power ultrasonic agitation at 125 W, for 10 min by using a Sonopuls HD3200 ultrasonic homogenizer from Bandelin (Berlin, Germany). A schematic of the synthesis route is shown in Figure 1 and the proportions of the reagents employed are compiled in Table 1.

The amount of n-octylamine and TiO_2_NPs with respect to the silica oligomer were 0.36% *v*/*v* and 1% *w*/*v*, respectively. The concentration of AuNPs was modified in the range 0–0.50% *w*/*w* (Au/TiO_2_ ratio). Since Au is added in an aqueous dispersion, the water content was also modified (see Table 1). The synthesized sols were designated ST$Au, where S and T indicate the presence of SiO_2_ and TiO_2_, respectively, $ is the ‱ *w*/*w* of Au/TiO_2_.

### 2.3. Sol-Gel Characterization

Immediately after the synthesis of the sols, their rheological properties were studied using a concentric cylinder viscometer (model DV-II+ with UL/Y adapter) from Brookfield (Middleborough, MA, USA). Experiments were performed at a constant temperature of 25 °C maintained by recirculated water from a thermostatic bath. A shear stress versus shear rate flow curve was generated.

Next, 3 mL of sols were deposited on plastic Petri dishes with a diameter of 85 mm and maintained at room temperature. The spontaneous sol-gel transition took place and the gels were dried at laboratory conditions until constant weight. The obtained xerogels were characterized according to the following procedures:

The UV-visible absorption spectra were recorded on a PG Instruments T92+ spectrophotometer (Leicestershire, UK) with an integrating sphere, and by using BaSO_4_ powder as white reference. The band-gap values were calculated by using the Kubelka-Munk function and the Tauc plot [58,59].

Textural characterization was performed by N_2_ Physisorption at 77 K, using a Quantachrome Autosorb IQ (Boynton Beach, FL, USA). The adsorption data were analysed using a hybrid NLDFT (non-local density functional theory) approach [60] that allows quantification of both micro- and mesopores in order to obtain the pore size distribution of materials containing pores of different geometry.

The previously described TEM/STEM microscope was used to characterize the structure of the materials under study. The samples were prepared by powdered deposition onto lacey carbon coated copper grids.

### 2.4. Application on Building Material and Characterization

A limestone commonly used in building was chosen. This stone presents a homogeneous structure, composed of a micritic matrix of calcite and has an open porosity of around 12%. The white color of this limestone is ideal for evaluating its color changes. The sols were applied directly (without dilution) on 5 cm × 5 cm × 2 cm samples of stones by spraying onto one of the larger faces until its saturation. The surfaces were maintained wet during 1 min and excess sols were removed by spraying air. Next, the samples were weighed to calculate the uptake of products. Finally, the treated stones were dried under laboratory conditions until their weights were constant (approximately two weeks) and their dry matters were calculated.

Next, we evaluated the changes in stone color induced by the treatments. This effect was determined by using a solid reflection spectrophotometer, Colorflex model, from HunterLab (Reston, VA, USA). The conditions used were: illuminant D65 and observer 10°. CIELa*b* color space was used and variations in color were evaluated using the parameter: total color difference (ΔE*) [61]. $\Delta E^{*}=\sqrt{\Delta L^{*}{}^{2}+\Delta a^{*}{}^{2}+\Delta b^{*}{}^{2}}$ where ΔL*, Δa* and Δb* are the differences for each colour coordinates. 

Scanning electron microscopy (SEM) images of the coatings were taken using a Nova NanoSEM model from the FEI Company (Hillsboro, OR, USA), working at an acceleration voltage of 3 kV.

We evaluated the thickness of the coatings using photographs of transversal cuttings of treated samples dyed with methylene blue for better observation. The images were taken using optical microscope Eclipse LV150 equipped with a camera DS-Fi1, both from Nikon (Minato, Japan). 

The adherence of the coating to the stone surface was evaluated by performing a peeling test using Scotch^®^ MagicTM tape (3 M) (Maplewood, MN, USA). The test was carried out by sticking a piece of adhesive tape on the sample surfaces and determining the increase of the tape weight after it is detached, in accordance with a previously reported method [62].

The test for evaluating the self-cleaning activity of the materials under study was adapted from a standard procedure [63]. Firstly, 0.5 mL of a 1 mM solution of methylene blue (MB) in ethanol was deposited, drop by drop, on the treated faces of the samples and on their untreated counterpart. Next, the samples of stones were irradiated in a solar degradation chamber, Solarbox 3000eRH from CO.FO.ME.GRA. (Milan, Italy), equipped with a 2500 W xenon arc lamp and an outdoor UV filter. Incorporated detectors controlled and monitored the temperature, humidity and irradiance (in 300–800 nm range). The conditions in the chamber were 500 W/m^2^ of irradiance, 55 °C of temperature and 60 mg/m^3^ of absolute humidity. The evolution of color and diffuse reflection spectra with time were determined by using the previously described spectrophotometer. Recently it has been suggested that the photocatalytic degradation of dyes undergo by a sensitization mechanism and the dye would act as a visible light antenna [64,65]. However, we used MB because it is an excellent model to determinate how our coatings will remove coloured pollutants, which is one of the most challenging issues in building conservation.

We evaluated the complete oxidation of MB using a HiCube mass spectrometer from Pfeiffer (Asslar, Germany), employing a 10 mL/min of O_2_ (4%)/Ar flow, over a cylindrical (ø5 × 2 cm) stone piece treated with photocatalytic coating and stained with MB. The staining procedure was the same for self-cleaning test. The sample was placed in AISI316 stainless steel reactor (in-house design) over a bed of quartz powder in order to reduce the void volume. Evolution of CO_2_ (*m*/*c* = 44) and H_2_O (*m*/*c* = 18) in the gas phase versus time were recorded. After the stabilization period the sample was irradiated with artificial solar light from 300 W Ultra-Vitalux lamp from Osram (Berlin, Germany) located 20 cm above the sample. We used a gas chromatograph Trace 1310 from Thermo Scientific (Waltham, MA, USA) equipped with a pulse discharge detector and a Carboxen 1010 PLOT capillary column from Sigma-Aldrich (St. Louis, MO, USA) to determine the CO_2_ concentration in the gas flow from the reactor. In this way, we related the mass spectrometer signal with the CO_2_ concentration. 

We carried out a self-cleaning test by using soot, a common staining agent of building facades. For soot deposition, according to a previous procedure [66], we exposed the treated faces of stones to the flame of a tealight for 30 s, after, we removed the excess and not adhered soot layer using compressed air. Next, the stone samples were irradiated in the solar degradation chamber and the evolution of color and diffuse reflection spectra were measured. The operation conditions employed in these photo-catalytic tests were identical to those used in the case of the previously described methylene blue degradation tests.

The hydrophilic properties of the samples were determined by the water contact angle test, using the sessile drop method. The measurements were obtained employing a commercial video-based, software-controlled contact angle analyser (OCA15plus, Dataphysics Instruments, Filderstadt, Germany). The test was carried out before and after light exposure in the solar box, by using the same conditions previously described during 24 h, in order to evaluate the induced hydrophilia phenomenon.

The ability of water to remove stains deposited on the samples was evaluated by the following experiment: a droplet composed of a mixture of terracotta powder and olive oil, simulating a greasy stain, was deposited on the samples. Next, they were subjected to a water stream (to simulate rain action) for 5 s.

## 3. Results and Discussion

### 3.1. Sol-Gel Characterization

All the sols showed a nearly Newtonian behaviour in the shear range evaluated. Thus, the viscosities were calculated as the slope of shear stress vs. shear rate curves. The viscosity values obtained for synthetized sols are presented in Table 2.

ST38Au and ST50Au sols were very viscous and they instantaneously gelled. Thus, the viscosity could not be measured. The viscosity values obtained for the other sols were similar to those corresponding to commercial silica sols employed for protecting building materials (i.e., Tegovakon V100 from Evonik (Essen, Germany), one of the most popular commercial stone consolidants, has a viscosity of 5.25 mPa·s at 25 °C) [16]. It confirms their suitability to be applied by common procedures, such as spraying, brushing, as coatings of building materials, even under outdoor conditions. In addition, a higher viscosity restricts the sol penetration into the substrate porous structure, this imply the reduction of adhesion between coating and substrate.

We observed two trends in the rheological behaviour of the sols under study. Firstly, as we previously observed [20], the inclusion of TiO_2_NPs in the sols increased their viscosities. Secondly, we observed a clear direct correlation between viscosity and the gold aqueous dispersion content in the sols. It is well known that the hydrolysis rate of the sol-gel reaction is increased by water concentration [67]. The higher hydrolysis rate produces a higher overall progress of the sol-gel reaction and thus, the viscosity increases. 

The sols stored in a closed bottle were maintained as sol for at least 6 months, whereas the sols deposited on plastic Petri dishes gel spontaneously, giving rise to crack-free and homogeneous xerogels. During this sol-gel transition, *N*-octylamine plays several roles: (1) it acts as a basic catalyst of the sol-gel reaction [21]; (2) it promotes formation of a particulated silica mesostructure, preventing cracking [21] and (3) it adjusts the pH of the media to values above the isoelectric point of TiO_2_ NPs, giving rise to negative charges in these NPs that produce mutual repulsive forces. Thus, TiO_2_ aggregation is avoided during sol-gel transition and, consequently, a homogeneous gel is produced [21,68,69].

Regarding the gel time, two factors are key. Firstly, the addition of TiO_2_NPs reduced the gel time due to the TiO_2_NPs acting as seeds, promoting an early nucleation of SiO_2_NPs around TiO_2_ species and the subsequent faster gel growth [70]. Secondly, the increase of gold aqueous dispersion content also produced a significant decrease in the gel time. In this case, an increase in the hydrolysis rate, due to the higher water content, is responsible for this phenomenon [67]. 

Gel time is also a key parameter for building materials application, especially when they must be applied in situ, because instantaneous gel times prevent sol penetration into the porous structure of the substrate. Therefore, we discarded the xerogels with higher Au content (ST38Au and ST50Au) due to their previously reported short gel time and high viscosity.

**Figure 2:**
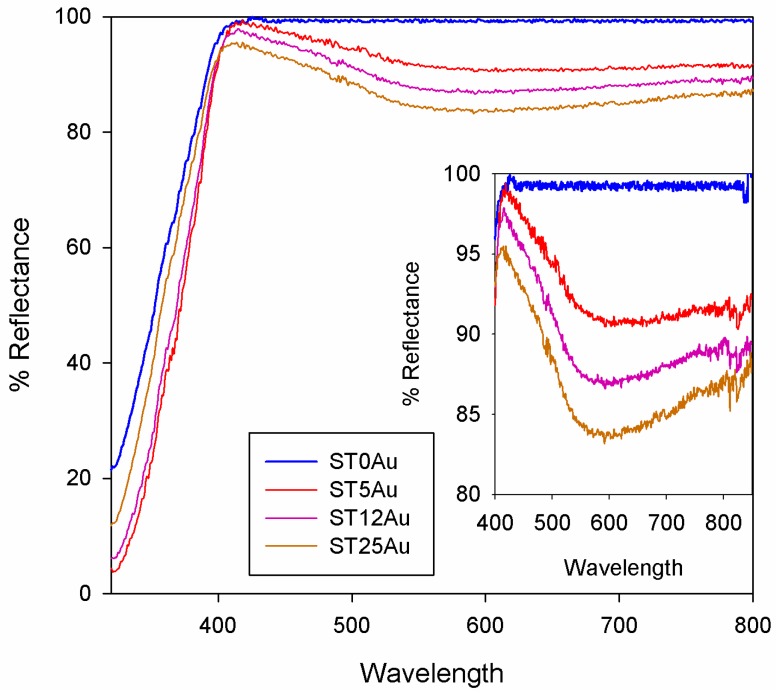
Reflectance UV-Vis spectra of powdered photocatalysts. Inset, detail of visible range.

Regarding the appearance of the xerogels, the gel without TiO_2_NPs was transparent and those with TiO_2_NPs were opaque (ST0Au was white, and gels with AuNPs were purple-blue). The purple-blue color was increased as Au content was raised, highlighting the absorption of visible light. This absorption was due to the LSPR band of AuNPs [39], which was clearly visible in the UV-Vis diffuse reflectance spectra (Figure 2), whereas the photocatalyst without Au (ST0Au) did not present any absorption in the visible range. We also calculated the band gap of the photocatalysts from reflectance spectra and we obtained a band gap of around 3.25 eV for all the materials prepared, close to the TiO_2_ anatase band gap (3.2 eV). 

**Figure 3:**
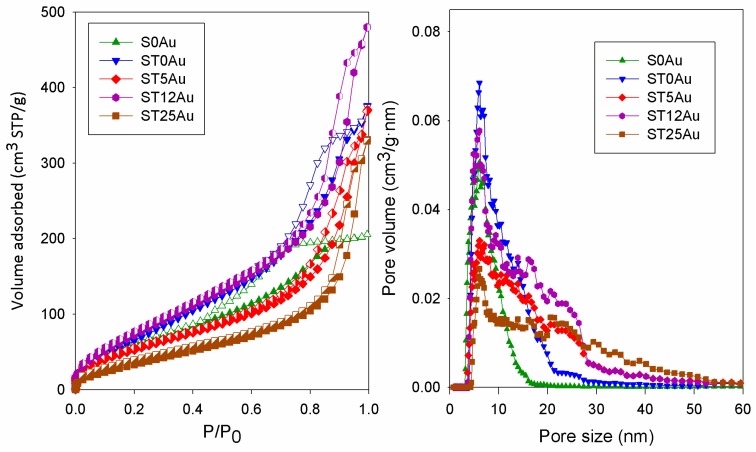
Isotherms and NLDFT (non-local density functional theory) pore size distributions obtained for the photocatalysts under study.

In order to investigate the porous structure of these materials, N_2_ physisorption tests were carried out. Adsorption–desorption isotherms and NLDFT pore size distributions, obtained from the adsorption branches for the materials under study, are shown in Figure 3. The textural data are compiled in Table 2. All materials show type IV(a) isotherms [71], characteristic of mesoporous materials due to the role played by n-octylamine [48]. Other similar TiO_2_/SiO_2_ composites prepared previously in our laboratory presented similar isotherms [20,21,38].

Regarding the hysteresis, S0Au (without TiO_2_NPs and without AuNPs) had a triangular hysteresis loop classifiable as H2(b). ST0Au (with TiO_2_NPs but without AuNPs) had a hysteresis with an elongated triangular shape, an intermediate situation between H1 and H2. The materials with gold had a H1 hysteresis (characterized by parallel and vertical branches). Hysteresis types H1 and H2 are characteristic of particulate materials formed by aggregation of spherical SiO_2_ particles. H1 presents a better connectivity between pores than the H2 isotherm [71,72]. In conclusion, the trend observed in the xerogels under study indicates that the addition of aqueous dispersions of TiO_2_NPs and AuNPs increase the interconnectivity of the pores of the original SiO_2_ matrix. In the case of AuNPs, we think the increase in porous connectivity was produced by the water increase, since the NPs are inert and their low concentration cannot modify the material structure. In order to confirm the water effect, we prepared xerogels equivalent to ST5Au, ST12Au and ST25Au by replacing aqueous dispersion of AuNPs with water. We obtained isotherms very similar to those corresponding to the materials with AuNPs (Figure S1), confirming the role played by water in the mesostructure of the xerogels. 

All the materials presented similar pore size distributions with a maximum at 6 nm, and a tail fitting to larger sizes. We found the greatest differences in the width of the pore distributions. S0Au exhibited the narrowest distribution (≈3–16 nm). The addition of TiO_2_ caused the appearance of larger pores (≈4–30 nm), and materials with AuNPs further increased the pore size (≈3.5–55 nm). These results will be discussed together with the TEM data. In addition, the presence of larger pores can be responsible for an increase in pore connectivity and the subsequent transition of the previously described hysteresis shape.

Regarding the textural data, the addition of TiO_2_ increased the surface area and pore volume, whereas these parameters were reduced as AuNPs were added, except for ST12Au, which had the greatest surface area and pore volume of all the materials under study. The different proportions of water in these materials can be responsible for this trend because the water/alkoxysilane ratio strongly affects the structure of gels [73]. Specifically, Yu and Wang [74] prepared TiO_2_-SiO_2_ materials with different water contents and the following behaviours were proposed according to the water/alkoxide ratio: (1) Low molar H_2_O/alkoxysilane ratios, such as ST5Au, promote the partial hydrolysis of the precursor. Partially hydrolysed alkoxides condense, generating chain-like structures. The resulting cross-linked gel has a non-rigid structure, which can collapse easily during drying and ageing. Thus, the final structure has low pore volume and surface area; (2) High molar H_2_O/alkoxysilane ratios, such as ST25Au, promote the total hydrolysis of the precursor. In this case, the monomers condense to each other, creating a dense structure. This compact structure also has low pore volume and surface area; and, (3) Intermediate molar H_2_O/alkoxysilane ratios, such as ST12Au, cause a partial hydrolysis of the precursor greater than (1) and thus, more hydrolysable groups are available for condensation, giving rise to a reticulated 3D-structure. This type of structure is less compact than (2) but it is rigid enough to not collapse during the drying and ageing process. As result, the structure has the highest pore volume and surface area. 

STEM images of the synthesized AuNPs and their corresponding size distributions are shown in the supplementary information (Figures S2 and S3). They exhibited a significant anisotropy, with different shapes, and a particle size ranging from 1 to 30 nm with two maxima at 3 and 18 nm. The nanometric size of these particles is responsible of the LSPR effect observed by spectroscopy (see Figure 2).

**Figure 4:**
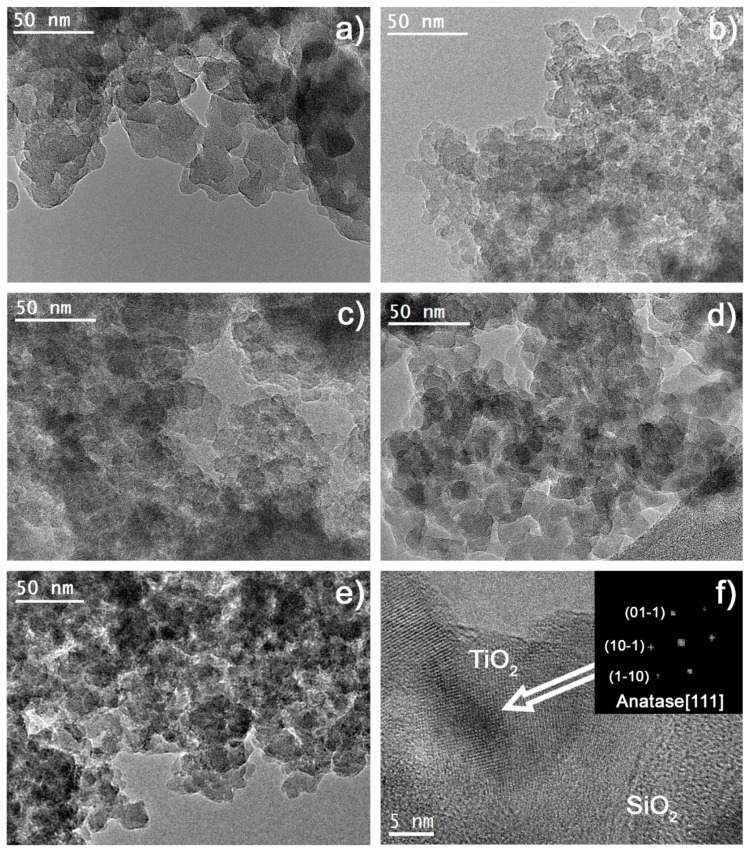
HRTEM images of photocatalysts (**a**) S0Au, (**b**) ST0Au, (**c**) ST5Au, (**d**) ST12Au, (**e**) ST25Au and (**f**) detail of a TiO_2_NP and its digital diffraction pattern.

HRTEM images of the Au-TiO_2_/SiO_2_ materials are shown in Figure 4. All of them were constituted by a silica matrix composed of amorphous silica particles, confirming the role played by n-octylamine [20]. However, the size of SiO_2_ particles was significantly different for the materials under study, ranging from 15 to 25 nm for the silica matrix (S0Au). The particle size was reduced as TiO_2_NPs were included in the formulation (10–15 nm for ST0Au) due to the nucleation promoted by TiO_2_ [70]. Thus, in the absence of TiO_2_ a larger SiO_2_ particle size is required for the nucleation process to begin. In the case of the materials with AuNPs, the particle size ranged from 10–20 nm. The water increased the hydrolysis rate of the silica precursor and, consequently, the condensation was faster, promoting an increased growth of silica particles [75].

The TiO_2_NPs were easily identified, because their crystalline structure was outlined against the amorphous silica matrix. The structure of the observed TiO_2_NPs was anatase (Figure 4f). However, AuNPs were not visible during the HRTEM observation, due to their low load into the materials and their overlapping of the SiO_2_ matrix.

The information obtained in physisorption experiments corroborated the obtained TEM images. Specifically, the packing of observed SiO_2_ particles produce interstitial holes that would correspond with the maximum pore size distribution observed in N_2_ physisorption (6 nm, see Figure 3). The TiO_2_NPs, with a size of around 25–45 nm, were considerably larger than the particles of the silica matrix and thus, they increased the size of interstitial holes. For this reason, ST0Au had a pore size distribution wider than S0Au (without TiO_2_). As AuNPs are integrated into the material, an open structure with greater pore size is observed (see Figure 3), as corroborated by N_2_ physisorption. ST12Au was clearly the less compact structure as the greater surface area and porous volume confirmed. 

**Figure 5:**
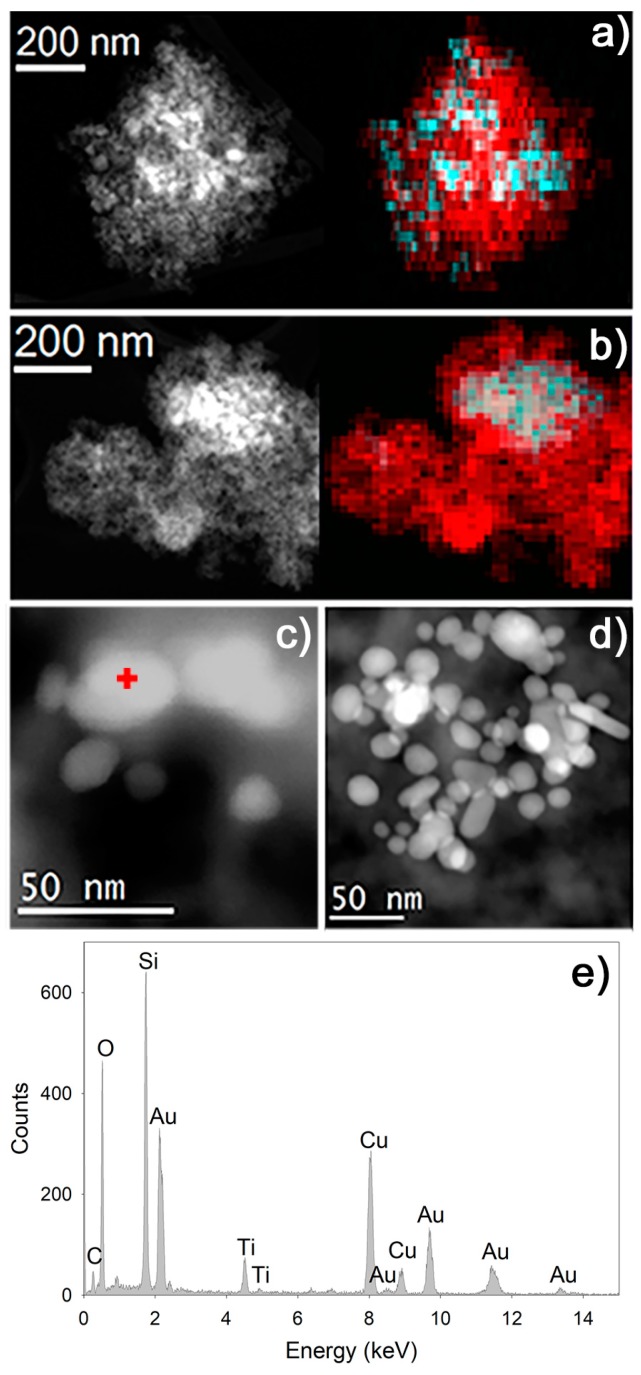
STEM-HAADF characterization of photocatalysts. (**a**) Image of ST12Au and its corresponding XEDS map for Si (red) and Ti (blue); (**b**) image of ST25Au and its corresponding XEDS map for Si (red) and Ti (blue); (**c**) AuNPs in ST12Au; (**d**) AuNPs in ST25Au and (**e**) example of XEDS point analysis acquired on the point marked in (**c**).

In STEM-HAADF (Figure 5) mode, we identify the distribution of components in the materials. Specifically, in ST12Au (Figure 5a), TiO_2_ was homogeneously dispersed throughout the matrix. However, in ST25Au (Figure 5b), TiO_2_ produced agglomerates of around 200 nm with a poor dispersion in the silica matrix. These distributions confirm that the TiO_2_ granulates (medium size of 20 μm) were disaggregated during the sol synthesis.

We also observe the AuNPs, because gold has a high contrast in this image mode. The size of these NPs (10–30 nm) matches the largest size (17.7 ± 4.4 nm) observed in the aqueous dispersion (see Figures S2 and S3). It was not possible to find the smallest AuNPs (3 ± 0.8) due to their reduced size, overlapping with silica matrix or aggregation. In addition, we observed that the AuNPs had a tendency to agglomerate as gold concentration is raised. Specifically, in ST12Au (Figure 5c), we found accumulations of around ten NPs disposed close to each other, whereas ST25Au (Figure 5d) presented accumulations of up to 50 NPs. 

The XEDS point analysis over AuNPs (Figure 5e) confirmed their composition and, importantly, revealed the presence of titanium at the same area. Therefore, it can be concluded that AuNPs were located close to TiO_2_NPs. This is fundamental for producing the energy transfers between AuNPs and TiO_2_ by LSPR phenomena [76]. The charge injection mechanisms take place when the plasmonic nanoparticles and semiconductor are in direct contact with each other, allowing an effective transfer of electrons and holes [77]. On the other hand, when metal and semiconductor are nearby but they are separated by a non-conductive thin layer (silica in our case), energy transfers can take place through near-field electromagnetic and resonant photon-scattering mechanisms [78]. 

**Figure 6:**
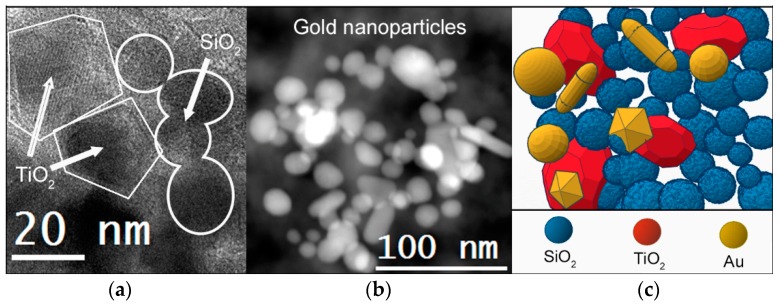
(**a**) HRTEM image of the xerogels under study; (**b**) HAADF image of detail of AuNPs into the xerogels and (**c**) model of nanostructure of photocatalyst built from these images.

According to the information obtained by the TEM study, a nanostructural model of the Au-TiO_2_/SiO_2_ materials synthesized in this study was proposed (see Figure 6). We can conclude that AuNPs with completely different morphology and size, and sharp crystalline TiO_2_ NPs with similar size are integrated in a mesostructured matrix composed of SiO_2_ particles, produced by the action of n-octylamine.

### 3.2. Application on Stone and Characterization

The sols synthesized in this study were sprayed, under laboratory conditions, onto the building limestone samples in order to investigate its photocatalytic behaviour and other properties associated with the coatings. 

Table 3 shows the uptake and dry matter values of the products. A clear relationship between the uptake and dry matter and the viscosity values (see Table 2) is observed. A lower viscosity allows a deeper penetration of the liquid product into the porous structure of the substrate and, consequently, produces higher uptake and dry matter. 

**Figure 7:**
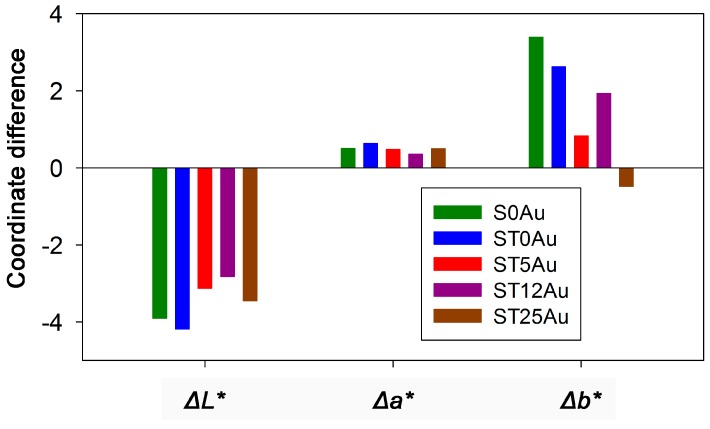
Changes in colour coordinates induced by the products under study.

One important limitation for practical application as building coatings would be changes in the colour of building materials. Therefore, total colour difference values (ΔE*) of the stone induced by the photocatalysts were measured and these results are given in Table 3. All the photocatalysts produced colour changes close to the generally accepted threshold value (ΔE* ≤ 5), even for the most restrictive applications such as ancient building restoration [79]. A digital reproduction of the stone colour, before and after the treatments, obtained from the CIEL*a*b* coordinates is shown in the supplementary information (see Figure S4). The most notable variation is observed for the products without AuNPs (S0Au and ST0Au). In order to analyse the contribution of each colour coordinate in ΔE*, the changes of the coordinates were examined separately (see Figure 7). The greatest changes were observed for *L* and *b* coordinates, corresponding to luminosity and red-green shift, respectively. Comparing the products, the most significant variations were observed for the *b* coordinate. The purple-blue colour of products with AuNPs is different to the green change produced by the S0Au and ST0Au treatments (The highest values of *b* coordinate). For this reason, the products with AuNPs produced the lowest colour changes.

The SEM images of the treated surfaces (Figure S5, in the supplementary information) show continuous and crack-free coatings. This confirms the role played by n-octylamine in preventing cracking of the xerogels, as previously discussed [80]. Also, we have measured the thickness of coating, it ranged from 3 to 12 µm, with an average of 8 µm, for all the samples. As an example, a representative photograph of the transversal cutting of treated sample is showed in Figure S6.

The values corresponding to the material removed by the peeling test are shown in Table 3. It was significantly reduced for the treated stones, their values being practically zero for all the products applied. These results confirm that all the products under study present a suitable adhesion to the stone and they even produce an effective consolidation of the limestone surface (the material removed is lower than that corresponding to the untreated limestone).

**Figure 8:**
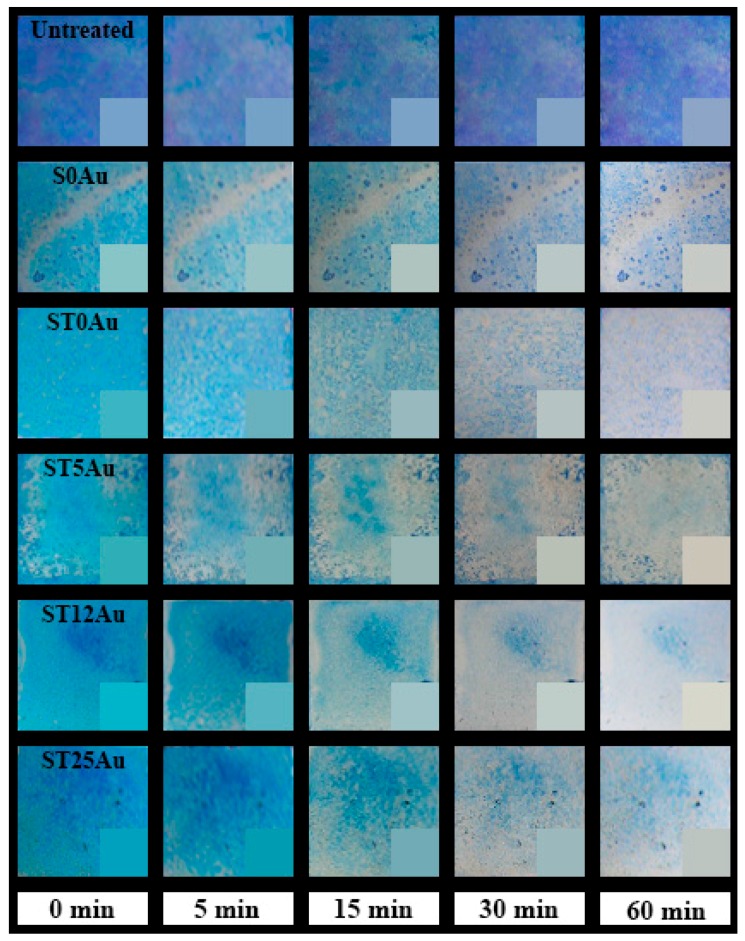
Photographs of evolution of decolourization of dye, deposited on the stone samples, during the first 60 min of the photodegradation test, the photographs correspond to the whole sample surfaces, 5 × 5 cm. The insets include the digital reproduction of the stone colour from the colour coordinates.

Finally, and most importantly, we evaluated the self-cleaning effectiveness of the products under study by analysing the degradation of methylene blue (MB), deposited on the coated stone samples, under visible radiation. The evolution of dye degradation for the treated samples and their untreated counterparts is shown in Figure 8. In order to evaluate these differences, we recorded UV-visible absorbance spectra in the stained treated samples and their untreated counterpart (see Figure S7). In all the cases, we observe two maxima in the spectra at 660 and 600 nm that can be related to the presence of MB^+^ and (MB^+^)_2_, respectively [81]. The peak associated to (MB^+^)_2_ is higher in the case of the untreated sample, whereas the opposite effect is observed in the treated samples. The shift of the absorbed maximum to a lower wavelength explains the observed violet hues of the untreated samples [82,83]. On the other hand, the slight differences observed between the treated samples can be associated with different MB penetration due to the different coating properties (textural properties, coating penetration, etc.) and irregularities of the stone samples.

**Figure 9:**
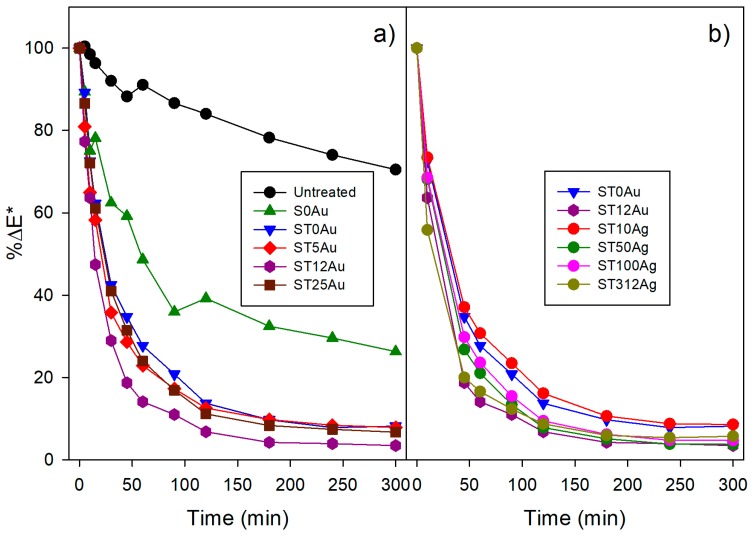
(**a**) Evolution of %ΔE* for the untreated and treated stone samples stained with methylene blue; (**b**) Comparison of results for ST and ST12Au, with the results obtained for the stones treated with different Ag-TiO_2_/SiO_2_ products.

In order to quantify the sample self-cleaning effectiveness, we use the total color difference, according the following equation:

(1) $$\%\Delta E^{*}=\frac{\Delta E^{*}}{\Delta E_{0}^{*}}$$

where $\Delta E^{*}$ is the total color difference at a specific time with respect to the sample before staining, and $\Delta E_{0}^{*}$ is the total color difference of the sample prior to starting the decolorization process. These results are presented in Figure 9.

The results included in Figure 9a show greater self-cleaning activity for the treated stone samples than the untreated stone. On the other hand, we want to comment that the MB degradation did not take place in dark conditions (see Figure S8). The untreated stone samples and their counterpart treated with the non-photoactive coating (S0Au) showed MB degradation. As reported in the literature [84], MB is degraded, under UV light, due to a purely photochemical mechanism associated with MB photolysis. In order to confirm MB photolysis, we have carried out a photodegradation test of MB deposited on quartz powder. Figure S9 in supplementary information shows the plot degradation and the evolution of methylene absorbance with the irradiation time.

The degradation rate of the sample treated with S0Au is significantly higher than that corresponding to the untreated sample due to the following issues:

- The degradation of the MB monomer (MB^+^), which is mainly adsorbed in the treated sample, is faster than that of the dimer (MB^+^)_2_ [85,86].
- The coating restricts the MB penetration into the substrate and it has a large surface area compared with the stone. Therefore, MB is more exposed to light, oxygen and humidity.
- The stone has a greater absorption in the UV range than the silica coating (see Figure S10). This reduces the amount of high energy light available to produce the MB photolysis.

Comparing the products, the treatments including TiO_2_ were more effective than those corresponding to S0Au, ST12Au being the most effective. This enhancement of self-cleaning activity is obviously due to the photocatalytic effect of TiO_2_.

Moreover, since one of the first objectives of this work is to improve the photocatalysis of the materials by using Au instead of Ag, in Figure 9b, the Au-TiO_2_/SiO_2_ coating with the best performance (ST12Au) is compared with previously developed Ag-TiO_2_/SiO_2_ treatments [38]. The Ag-based coatings showed a poorer performance than that corresponding to the Au-TiO_2_/SiO_2_ based coating with the best performance, and only the product with the highest Ag content (3.2%) showed a similar degradation to the best-performing Au-TiO_2_/SiO_2_ coating (with an Au content of 0.12%). Additionally, it should be pointed out that the 3.2% Ag-TiO_2_/SiO_2_ coating showed an increase in ΔE* for a degradation time above 180 min. This was due to the appearance of a brown colouration in the stone surface produced by the photochemical reaction of silver.

On the other hand, using the %ΔE* procedure, it was difficult to discern the activity sequence of the evaluated products because their degradation plots are overlapped. In spite of this methodology being commonly employed for evaluating the self-cleaning activity of building materials [10,11,18,46,50], their poor resolution led us to propose an alternative approach for self-cleaning testing on building materials.

Specifically, this approach is based on applying the Kubelka-Munk theory to the UV-Visible reflectance spectra. The Kubelka-Munk theory [87,88] is commonly used to explain the light absorption in a thin layer of dye deposited on a non or low-absorbent substrate [54], which can be considered to approximate the MB deposited on the stone samples under study. The following equation, specifically addressed for these systems is included in Equation (2):

(2) $$f\left(R_{\infty }\right)=\frac{{\left(1-R_{\infty }\right)}^{2}}{2R_{\infty }}=\frac{k}{s}$$

where $f\left(R_{\infty }\right)$ (hereafter KM) is equivalent to absorbance, $R_{\infty }$ is the diffuse reflectance, *k* is the dye molar absorption coefficient and *s* the surface scattering coefficient.

**Figure 10:**
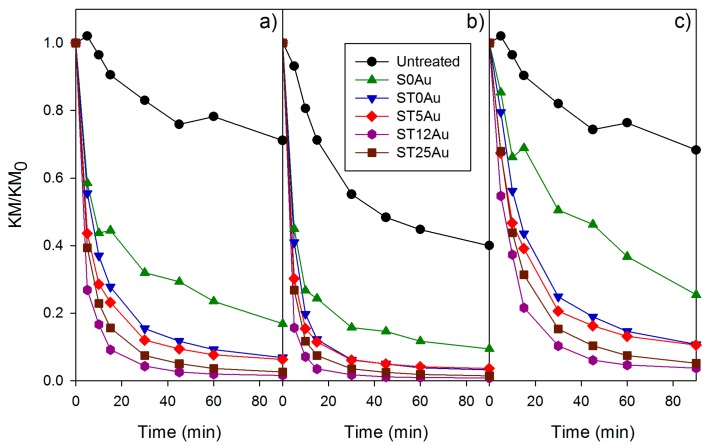
Evolution of KM/KM_0_ for the untreated and treated stone samples stained with methylene blue, (**a**) overall degradation; (**b**) monomer degradation and (**c**) dimer degradation.

From this equation, the experimentally obtained diffuse reflectance spectra can be converted into the corresponding absorbance spectra, which is directly related to the quantity of MB. Specifically, we used the evolution of KM/KM_0_, KM_0_ as being the initial absorbance in the absorption maximum, to determinate the decay of concentration of the MB over time. This method should be more precise in determining the self-cleaning activities of the products under study, because the absorbance is directly related to the quantity of dye. The plots (Figure 10a) were similar to those obtained by the popular space color coordinates method, but the difference in activity between the coatings was easily discernible and thus, the following order in the self-cleaning activity can be established:

ST12Au > ST25Au > ST5Au > ST0Au > S0Au > Untreated.

This trend can be explained as follows: ST0Au activity was greater than that of S0Au, confirming the photocatalytic effect of TiO_2_. All the products with AuNPs significantly increased the self-cleaning activity with respect to ST0Au. This demonstrates that the addition of AuNPs improves the photoactivity of TiO_2_ in the photocatalysts under study.

In addition, the use of absorbance spectra allows a deeper study of methylene blue degradation. Specifically, a specific wavelength to discern between the degradation of MB monomer (660 nm) and dimer (600 nm) can be studied. The degradation profile of the monomer (Figure 9b) and the dimer (Figure 9c) show the same trend as the overall degradation plot (Figure 9a), but the monomer degradation is considerably faster than dimer degradation. As previously reported [85], this analysis is important because monomer and dimer degradation are not comparable due to their different degradation rates. In this study, the treated samples trend was similar for the three degradation profiles because MB is mainly adsorbed as a monomer. However, the difference between the untreated samples and their counterpart treated with S0Au is significantly different for overall and dimer degradation profiles because MB, as previously discussed, corresponds to dimer and monomer species, respectively and thus, the degradation rate is different.

As MB absorbance is directly related to its concentration, we can use its evolution over time to fit the experimental data to a rate equation. The photodegradation of MB deposited on TiO_2_ coated glass has been reported as a first order kinetic process [85]. However, our results were not well adjusted to a first order rate equation. This can be related to: (1) the coating thickness, 200 nm and 8 µm for the previous work and our study, respectively; (2) the porosity of the substrate, glass (non-porous substrate) and stone (porous substrate). In order to confirm this hypothesis, the ST0Au coating was deposited on glass by dip coating, and was subjected to a MB degradation experiment under the conditions described in the experimental section. The MB degradation was perfectly adjusted to a unique first order process (see Figure S11), confirming this study’s hypothesis. A specific rate equation was obtained from the experimental results corresponding to the MB degradation plots, the initial and final values were independently fitted to two different first order equations. This can be associated with the existence of two processes with different rates, as previously proposed, for the degradation of dyes deposited on TiO_2_ [89].

The assumption is an extreme simplification of the two kinetic models proposed in a previous paper [89]. One of them, named “unexposed portion model”, assumes that only TiO_2_ directly exposed to light can be degraded, and that the dye portion that penetrates into the TiO_2_ layer (in the dark) cannot be degraded. Another alternative model, named “intensity influence model”, considers that light intensity decreases through the thickness of TiO_2_ and thus, the rate constant is dependent on the light intensity received. Both of these assumptions were combined and simplified to explain the two kinetics considered in the rate equation proposed in our work. Firstly, similar to the first model, the existence of two portions of MB was considered: (1) MB exposed on the sample surface, and, (2) MB into the substrate structure. Secondly, it was considered that the rate constant for MB inside the sample is reduced, due to its lower exposure to light. However, instead of considering a gradual rate constant decreasing through the penetration depth, a constant value is simplified which is lower than that corresponding to the surface degradation rate. Thus, the following equation combining two first-order processes was achieved:

(3) $$\frac{KM}{KM_{0}}=\left(1-x\right)e^{-k_{1}t}+xe^{-k_{2}t}$$

where *k*_1_ is the reaction rate of the fast degradation process (surface), *k*_2_ is the degradation of the slow degradation process (inside) and *x* is the fraction of MB degraded by means of the slow process. These kinetic parameters and the coefficient of determination (R^2^) are compiled in Table 4.

These obtained parameters showed a good fit to the measured values (see Figure S12). Thus, we can use them to make quantitative comparisons. According to the previous consideration, the *k*_2_ constants were effectively several orders of magnitude lower than *k*_1_. Using the *k*_1_ constants to compare the self-cleaning properties of the products under study, the activity sequence was in accordance with those obtained by the analysis of the evolution of KM/KM_0_ (see Figure 10), *k*_2_ also showed a similar trend. Comparing *k*_1_ for the monomer and dimer degradation, we observe that *k*_1_ is higher for the monomer, around three times that for treated stones, thus confirming the faster monomer degradation. Finally, we noticed differences in the *x* parameter values. S0Au treated stone presented a significant reduction of the *x* parameter with respect to untreated stone, as previously discussed; this is related to the presence of coatings preventing the MB penetration. The samples containing TiO_2_-based coatings had similar *x* values and they were lower than S0Au (without TiO_2_). This difference may be attributed to the different degradation processes that took place (photolysis and photodegradation).

We want to comment that the increase in photoactivity of the products did not present a direct relationship with the content of AuNPs, with ST12Au (intermediate content of AuNPs) being the most active product. We can explain this as a consequence of the influence of the structure and the texture of the coatings on their photoactivity. Photocatalyst structure is a relevant parameter, notably the Au and TiO_2_ distribution in the silica matrix [49]. ST12Au, with the best photoactivity performance, showed a homogeneous distribution of the photoactive components in the matrix, as observed by TEM (see Figure 5a). In the case of ST25Au (Figure 5b,d), a poor dispersion of Au and TiO_2_ into the silica matrix is observed.

Regarding the texture, since the dye degradation is a surface process, the contact between MB, TiO_2_ and the parameters acting in the redox process (light, O_2_ and H_2_O) need to be maximized [90,91,92,93]. Thus, photocatalyst surface area plays a key role in the MB degradation [92,94,95,96]. Pore volume is also a relevant parameter because diffusion of substances (O_2_, H_2_O and dye) to active photocatalyst sites occurs through the pore structure [20,91,97,98]. Thus, although ST25Au had the highest content of AuNPs, its low surface area and pore volume values (see Figure 3 and Table 2) resulted in its lower photoactivity. ST12Au with a medium content of AuNPs presented the highest surface area and pore volume in the products under study. Thus, ST12Au showed the highest photoactivity because it has the best compromise between (1) content of AuNPs, and consequent visible light absorption, (2) dispersion of TiO_2_ and AuNPs in the silica matrix and (3) textural parameters. 

**Figure 11:**
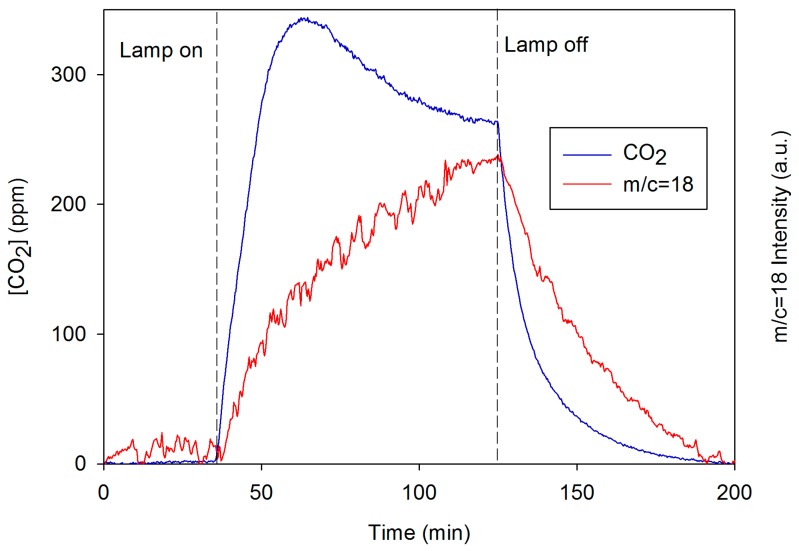
Evolution of CO_2_ concentration and *m*/*c* = 18 signal (water) during the mass spectroscopy experiment.

Finally, we confirmed that under the conditions of our assay the MB photodegradation took place via oxidation. The mass spectroscopy experiments (Figure 11) showed an evident CO_2_ and H_2_O liberation during the light irradiation of the ST12Au sample. We selected this sample because it presented the highest photoactivity, and thus, it should release the highest amount of CO_2_. When the illumination started, the CO_2_ concentration in air quickly increased and reached a maximum level of around 340 ppm after approximately 30 min, then the CO_2_ level slowly decreased. This behaviour is related to the faster MB degradation that we observed in the first min of the self-cleaning test. When the illumination was finished, the CO_2_ concentration began to decrease abruptly, confirming that the CO_2_ formation was interrupted. The water signal (*m*/*c* = 18) showed a similar behaviour, but its release profile was different, probably due to the water being adsorbed in the sample. The air mass spectrum during the illumination (see Figure S13) did not show signals in the range of mass/charge 50–185, corresponding to volatile organic compounds, which are intermediates of MB degradation. This evidence demonstrated that our photocatalysts have the capacity for oxidizing the MB completely to CO_2_ and water, and intermediate volatile organic compound are not released.

**Figure 12:**
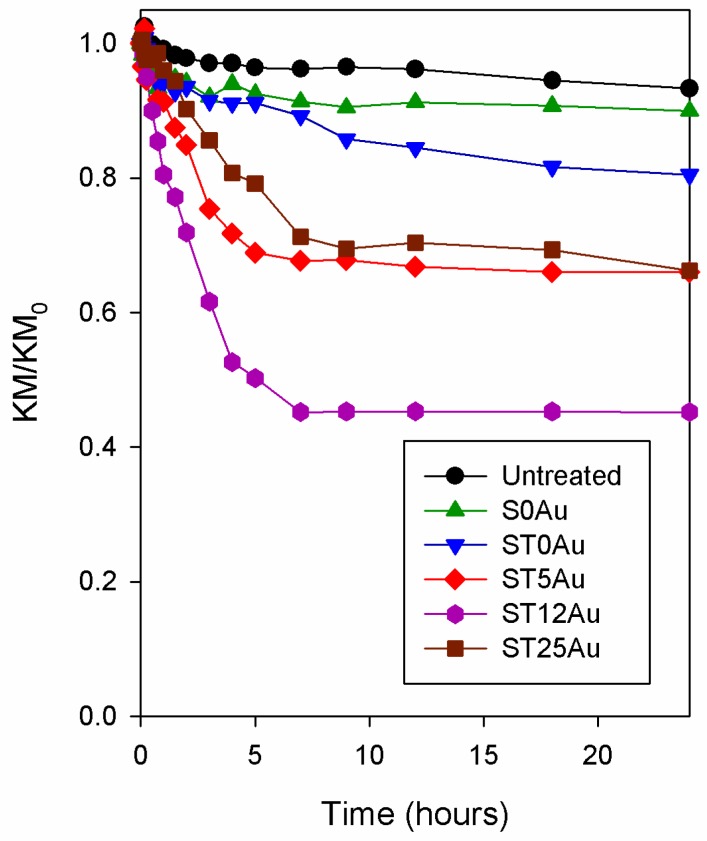
Evolution of KM/KM_0_ for the untreated and treated stones stained with soot.

In addition, we checked the degradation efficiency of the photocatalysts under study against a real staining agent for buildings. The self-cleaning test was carried out by using stone samples stained with soot. As a maximum absorbance in the visible spectrum is not observed for soot, we used the average KM values obtained in the spectral range of 420–440 nm to build the degradation plots. We chose these wavelengths because the maximum absorbance decrease took place in that zone of the spectrum (see Figure S14), and the average of several points corrects the noise of the measure. The soot decolourization trends (Figure 12) were similar to those obtained by MB: ST12Au > ST25Au ≈ ST5Au > ST0Au > S0Au > Untreated. By comparing soot and MB activity, we observed a significantly slower decolourization for soot. A fast soot decolourization was observed in the first 6 h of the test, but it did not progress for longer times. This was probably related to the soot penetration into the pore structure of the stone and its strong adherence to the substrate.

Finally, in order to evaluate the existence of photoinduced hydrophilicity on the samples under study, the water contact angle (CA) measurement was carried out on the surface of the samples, before and after light exposure. Before light exposure, all the treated surfaces showed hydrophilic properties, due to the hydrophilic nature of the silica matrix. Specifically, the untreated sample absorbed water and thus, the CA could not be measured. The CA values for the treated samples without TiO_2_ (S0Au) showed a medium value of 71° ± 8°. The incorporation of TiO_2_ into the coating increased the hydrophilic behaviour of the treated surface and thus, all samples containing TiO_2_ showed contact angles with a medium value of 44° ± 9°. The test was repeated 24 h after light exposure, showing similar CA values, probably due to the low TiO_2_ content in the coatings. 

**Figure 13:**
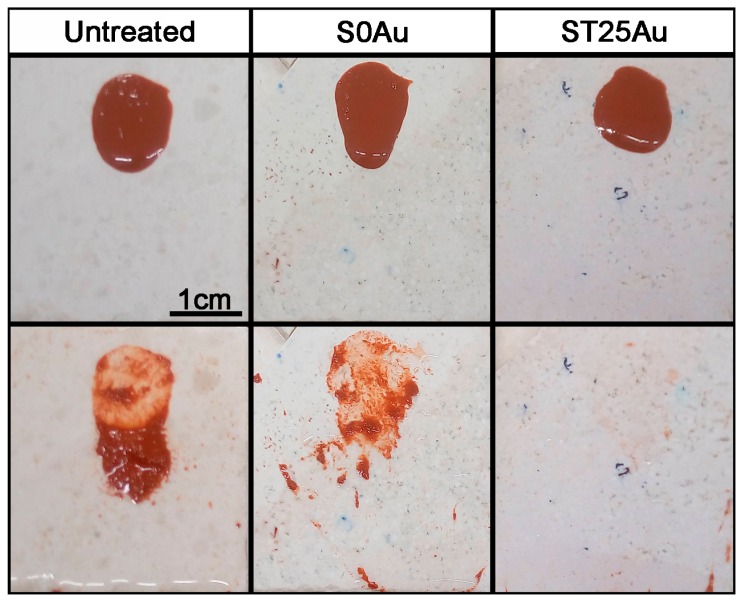
Photographs of the samples before and after the water streaming: untreated, S0Au and ST25Au. all photographs have the same scale.

In spite of the absence of induced superhydrophilicity, all the TiO_2_-based coatings showed an effective self-cleaning performance under water action, as shown in Figure 13, and in the video in the supplementary material. In the untreated sample, the water could not remove the stain droplet because it penetrated into the stone pores and thus, it was adhered. All the coatings prevented the stain penetration into the stone pores. In addition, the hydrophilic nature of the coatings promotes water spreading, favouring stain removal. Thus, the stain was only partially removed in the sample with the lowest hydrophilicity (S0Au), and it was completely removed in the other samples. 

## 4. Conclusions

We have designed a new and simple sol-gel route for producing Au-TiO_2_/SiO_2_ photocatalysts with application as self-cleaning coatings on building materials. The addition of a low content of AuNPs into the starting sol improves the TiO_2_ photoactivity under solar radiation, because a significant absorption in the visible range (45% of solar radiation) is promoted. The sols obtained by this route were applied on a common building stone, producing coatings, which adhere firmly to the stone and preserve their aesthetic qualities.

We compared two different methodologies for evaluating self-cleaning on building materials. The commonly employed color method, based on the study of space color coordinates, and an alternative absorbance method, based on the Kubelka-Munk theory. This comparison demonstrated that the color methodology has a limited applicability whereas the absorbance method shows clear advantages: (1) it allows differences between coatings to be clearly discerned; (2) it allows degradation of different dye species in the system to be studied independently (in this case MB dimer and monomer); and, (3) it allows fitting the experimental data to a rate equation that allows dye degradation in a porous substrate to be explained.

Finally, we established that the self-cleaning effectiveness of the photocatalysts under study did not present a direct relationship with the content of AuNPs, an intermediate Au content being the optimal value. We can explain this as a consequence of the influence of the structure and the texture of the coatings on their photoactivity. Specifically, the product with intermediate Au content showed the highest pore volume, the highest surface area and the best dispersion of the photoactive components.

## Figures and Tables

**Table 1:** Composition of the prepared products.

Product	% TiO_2_^a^	% Water Content ^b^	% Au/TiO_2_^c^
S0Au	0	0	0
ST0Au	1	0	0
ST5Au	1	0.33	0.05
ST12Au	1	0.83	0.12
ST25Au	1	1.67	0.25
ST38Au	1	2.50	0.38
ST50Au	1	3.33	0.50

^a^ %(*w*/*v*) TiO_2_/silica oligomer; ^b^ %(*v*/*v*) water/silica oligomer; ^c^ %(*w*/*w*) Au/TiO_2_.

**Table 2:** Viscosity and gel time of synthetized sols and textural properties of xerogels obtained.

Product	Viscosity (mPa·s)	Gel Time	S_total_ (m^2^/g)	V_pore_ (cm^3^/g)
S0Au	4.61	24 h	240	0.32
ST0Au	5.43	12 h	316	0.58
ST5Au	5.80	8 h	217	0.57
ST12Au	6.57	6 h	329	0.74
ST25Au	8.37	3 h	155	0.51
ST38Au	*	5 min	#	#
ST50Au	*	1 min	#	#

* Fast gelation, measurement impossible # material not studied.

**Table 3:** Averages values and standard deviations for properties of treated stones.

Sample	Uptake (g/m^2^)	Dry Matter (g/m^2^)	Δ*E* *	Peeling Test (mg/cm^2^)
Untreated	-	-	-	0.079 ± 0.014
S0Au	480 ± 60	258 ± 40	5.2 ± 0.6	0.019 ± 0.016
ST0Au	428 ± 60	246 ± 40	5.0 ± 0.3	0.011 ± 0.015
ST5Au	381 ± 90	203 ± 60	3.5 ± 0.6	0.021 ± 0.015
ST12Au	302 ± 90	171 ± 60	3.4 ± 0.5	0.016 ± 0.018
ST25Au	281 ± 60	153 ± 40	3.5 ± 0.6	0.019 ± 0.013

**Table 4:** Parameters of kinetic models calculated by the kinetic model proposed in this paper.

Sample	Degradation	*k*_1_ (min^−1^)	*k*_2_ (min^−1^)	*x*	R^2^
Untreated	Overall	0.030	1.59 × 10^−3^	0.812	0.964
	Monomer	0.039	1.33 × 10^−16^	0.620	0.991
	Dimer	0.027	1.95 × 10^−3^	0.798	0.966
S0Au	Overall	0.124	6.31 × 10^−3^	0.345	0.959
	Monomer	0.185	9.85 × 10^−3^	0.223	0.981
	Dimer	0.061	9.19 × 10^−3^	0.613	0.993
ST0Au	Overall	0.163	1.67 × 10^−2^	0.259	0.999
	Monomer	0.207	1.38 × 10^−2^	0.095	0.999
	Dimer	0.071	5.72 × 10^−3^	0.185	0.995
ST5Au	Overall	0.265	2.22 × 10^−2^	0.282	0.996
	Monomer	0.309	1.95 × 10^−2^	0.132	0.999
	Dimer	0.105	9.84 × 10^−3^	0.244	0.998
ST12Au	Overall	0.437	5.54 × 10^−2^	0.246	0.998
	Monomer	0.471	5.30 × 10^−2^	0.094	0.999
	Dimer	0.136	1.87 × 10^−2^	0.152	0.997
ST25Au	Overall	0.269	3.34 × 10^−2^	0.234	0.999
	Monomer	0.322	3.16 × 10^−2^	0.107	0.999
	Dimer	0.099	1.14 × 10^−2^	0.148	0.999

## Supplementary Materials

The following are available online at http://www.mdpi.com/2079-4991/8/3/177/s1, Figure S1. Nitrogen physisorption isotherms and pore distribution of photocatalysts with 1% of TiO_2_ prepared with different water content, Figure S2. HAADF-STEM image of AuNPs nanoparticles employed, Figure S3. Size distribution of AuNPs employed, Figure S4. Digital reproduction of stone color after and before treatments, Figure S5. SEM images of coated stones under study and their untreated counterpart, Figure S6. Optical Microscopy photograph of transversal cutting of treated stone. The coating was dyed with methylene blue for better observation, Figure S7. UV-visible absorbance spectra of MB deposited on untreated and on a treated stone, ST12Au treated sample was selected as representative example. The absorbance spectra were obtained from respective reflectance spectra, Figure S8. Evolution of %Δ*E** for a ST12Au treated stone sample stained with methylene blue maintained in dark conditions, Figure S9. Degradation plot and absorbance evolution for MB with the irradiation time, Figure S10. UV-visible absorbance spectra of S0Au coating and the limestone employed in this work. The absorbance spectra were obtained from respective reflectance spectra, Figure S11. Left, evolution of MB degradation on ST0Au coating deposited on glass. Right, its corresponding fitting to first order rate equation. Figure S12. Fitting between the KM/KM_0_ values measured (dots) and calculated by fitting to rate equation (lines) for the different degradations processes, overall (black), monomer (red) and dimer (blue), Figure S13. Mass spectrum of air from reactor obtained during the mass spectroscopy experiments when the sample was being irradiated with light, Figure S14. Example of soot absorbance spectra during soot self-cleaning test for ST12Au treated stone, Video 1. Self-cleaning test by water action.
