# Supplementary material for: TiO2-SiO2 Coatings with a Low Content of AuNPs for Producing Self-Cleaning Building Materials
Source: Nanomaterials (Basel). 2018 Mar 20;8(3):177. doi: 10.3390/nano8030177 (PMC5869668; doi:10.3390/nano8030177)
Supplement: Supplementary file 1 [file nanomaterials-08-00177-s001.zip › Supplementary Figures.docx]

*Supplementary Materials*

**TiO_2_-SiO_2_ Coatings with a Low Content of AuNPs for Producing Self-Cleaning Building Materials**


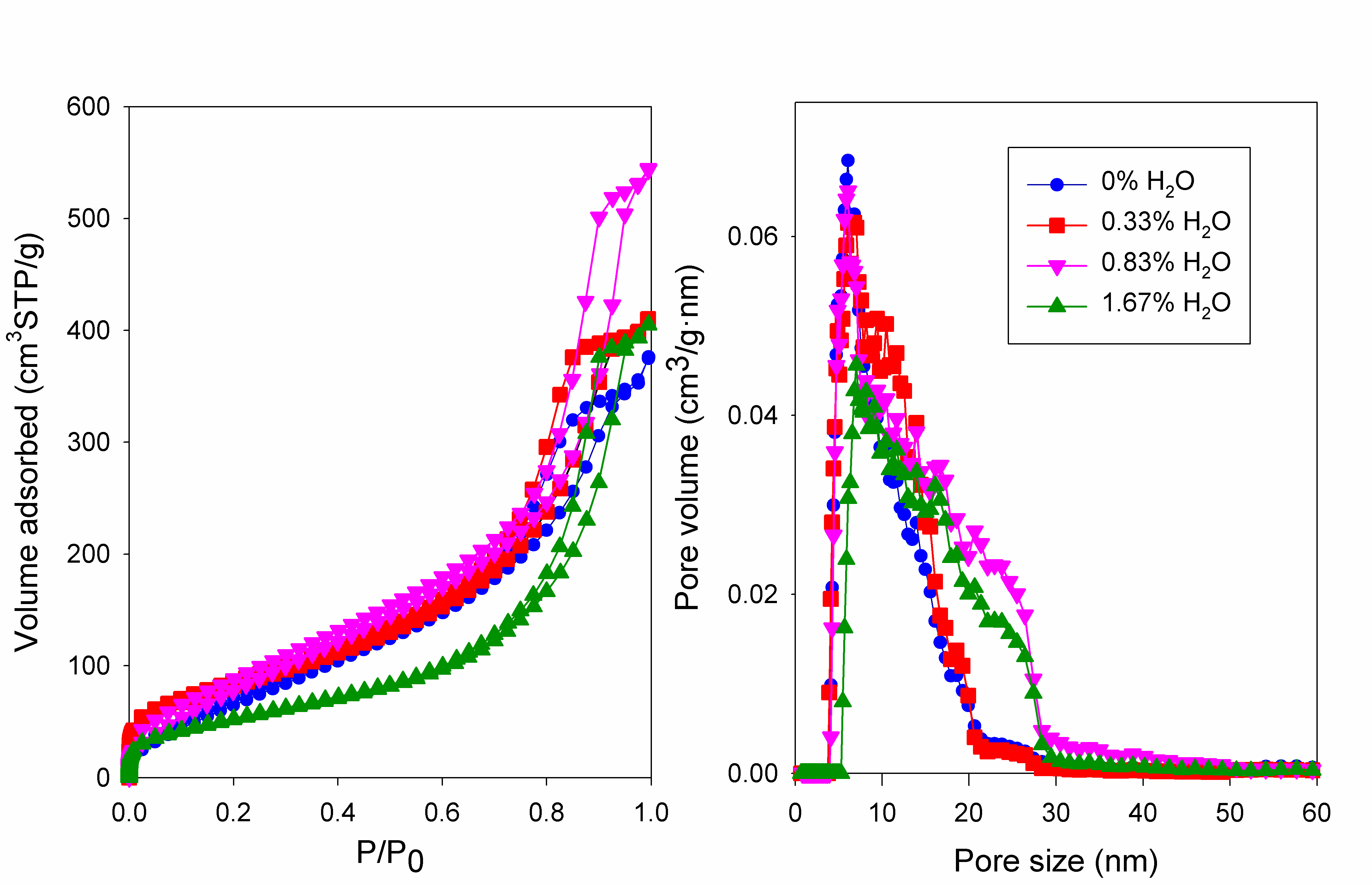


**Figure S1.** Nitrogen physisorption isotherms and pore distribution of photocatalysts with 1% of TiO_2_ prepared with different water content.


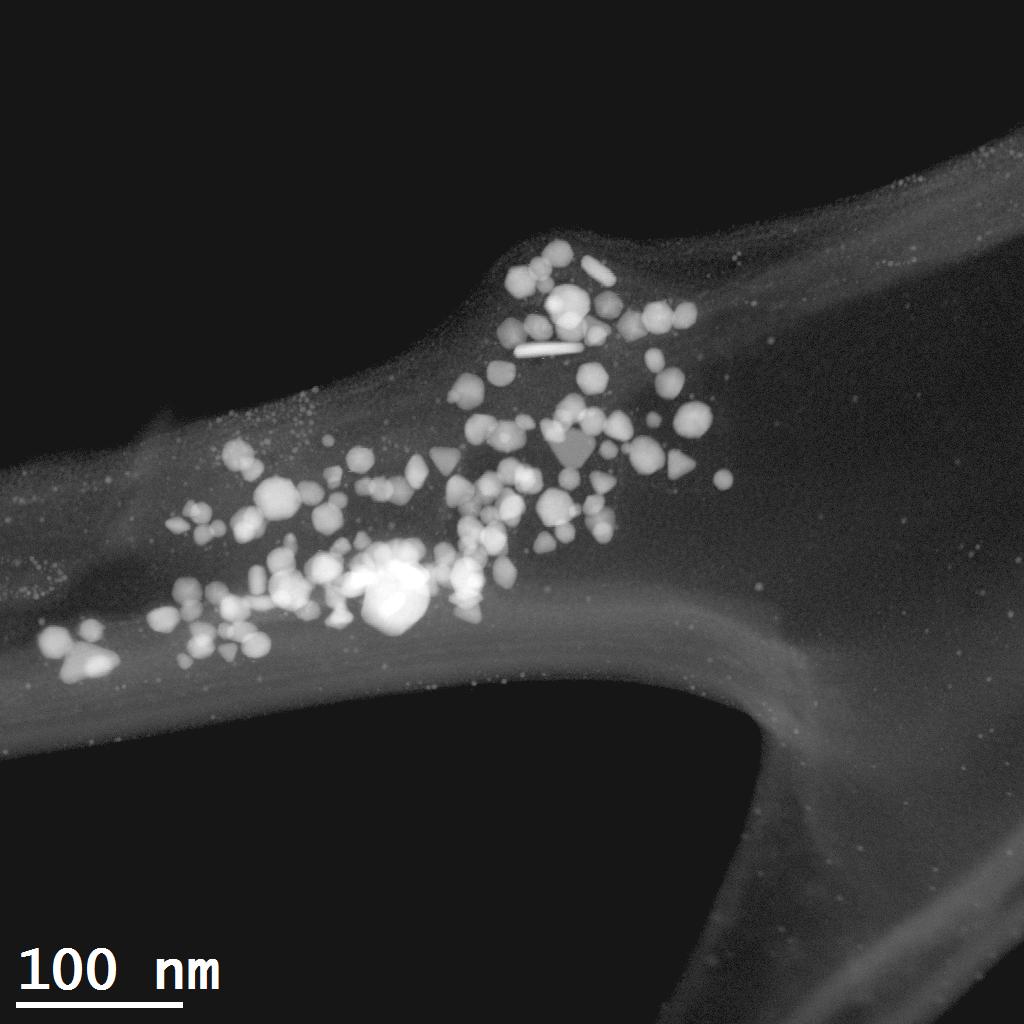


**Figure S2.** HAADF-STEM image of AuNPs nanoparticles employed.


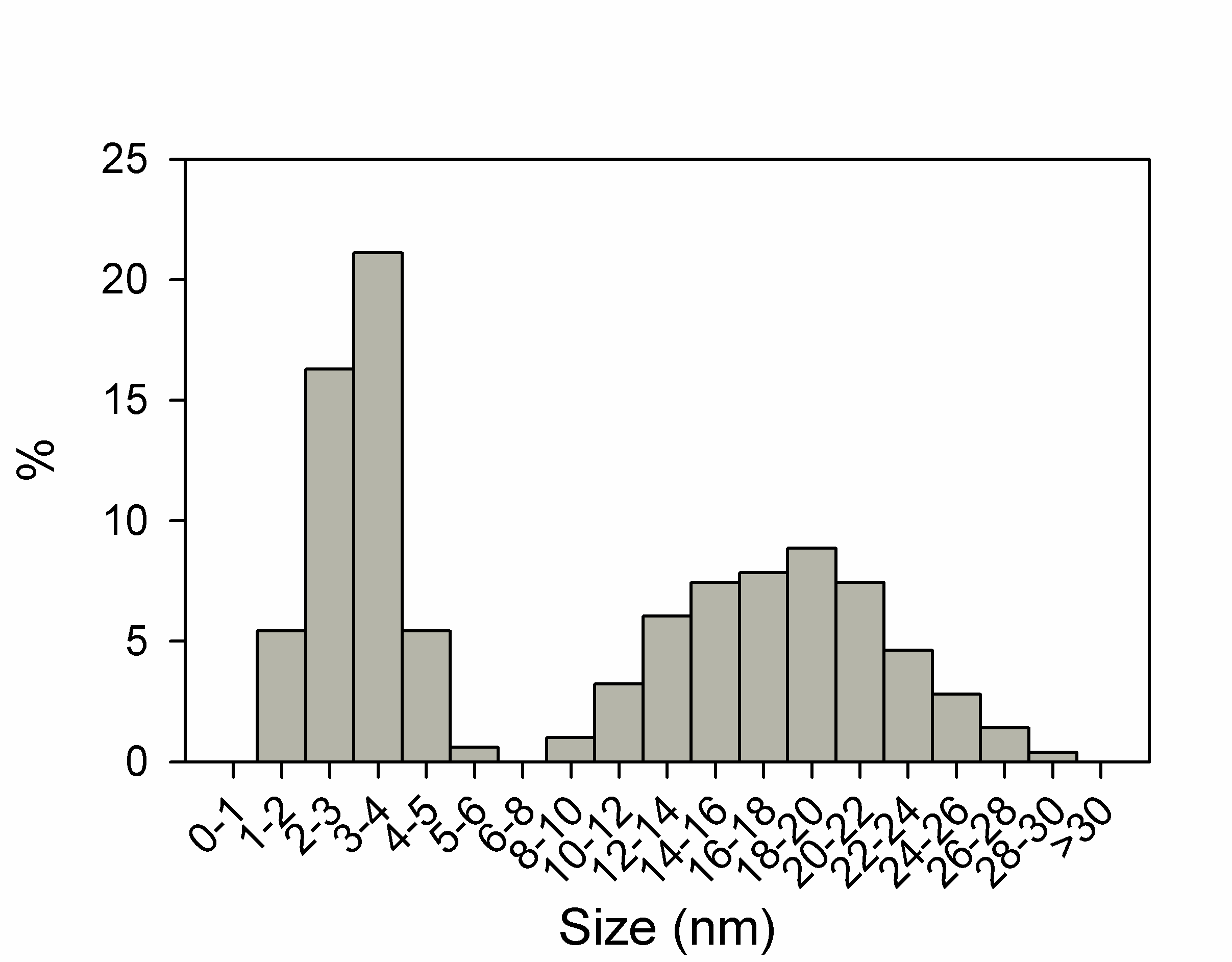


**Figure S3.** Size distribution of AuNPs employed.


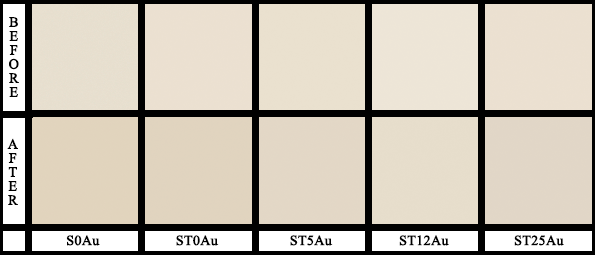


**Figure S4.** Digital reproduction of stone color after and before treatments.

**
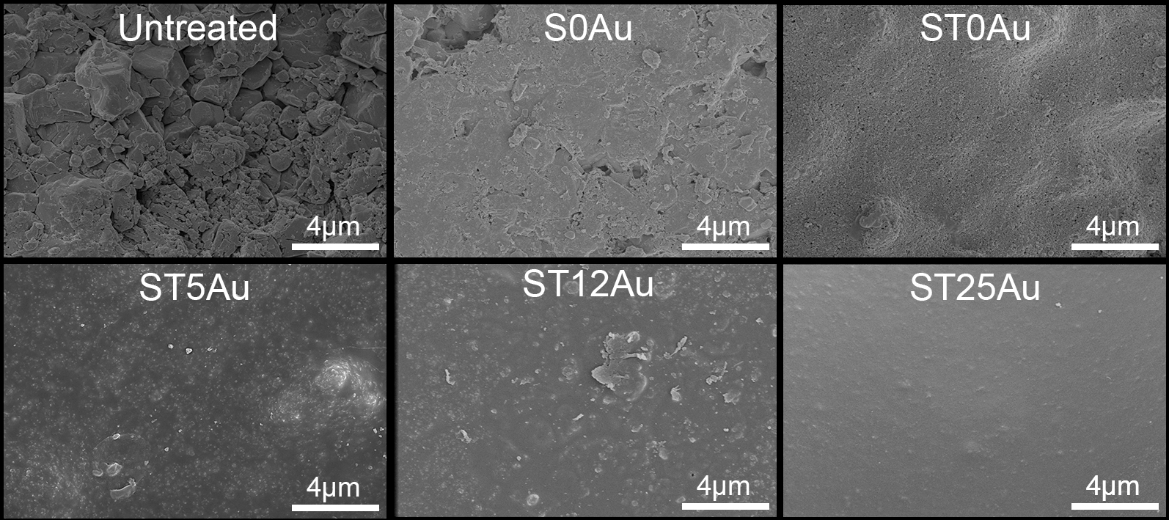
**

**Figure S5.** SEM images of coated stones under study and their untreated counterpart.


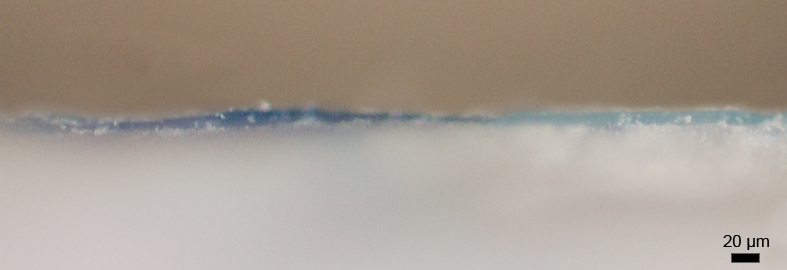


**Figure S6.** Optical Microscopy photograph of transversal cutting of treated stone. The coating was dyed with methylene blue for better observation.


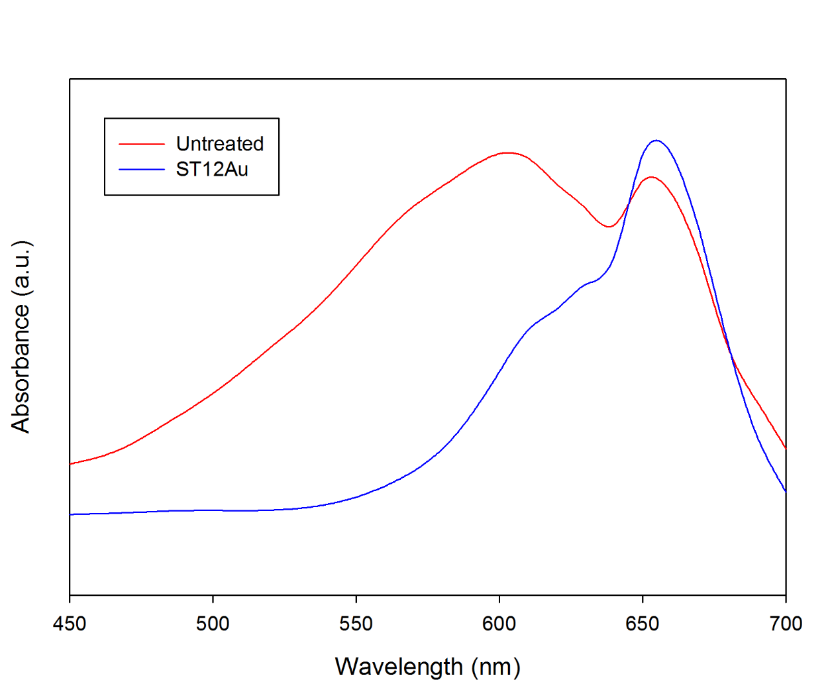


**Figure S7.** UV-visible absorbance spectra of MB deposited on untreated and on a treated stone, ST12Au treated sample was selected as representative example. The absorbance spectra were obtained from respective reflectance spectra.


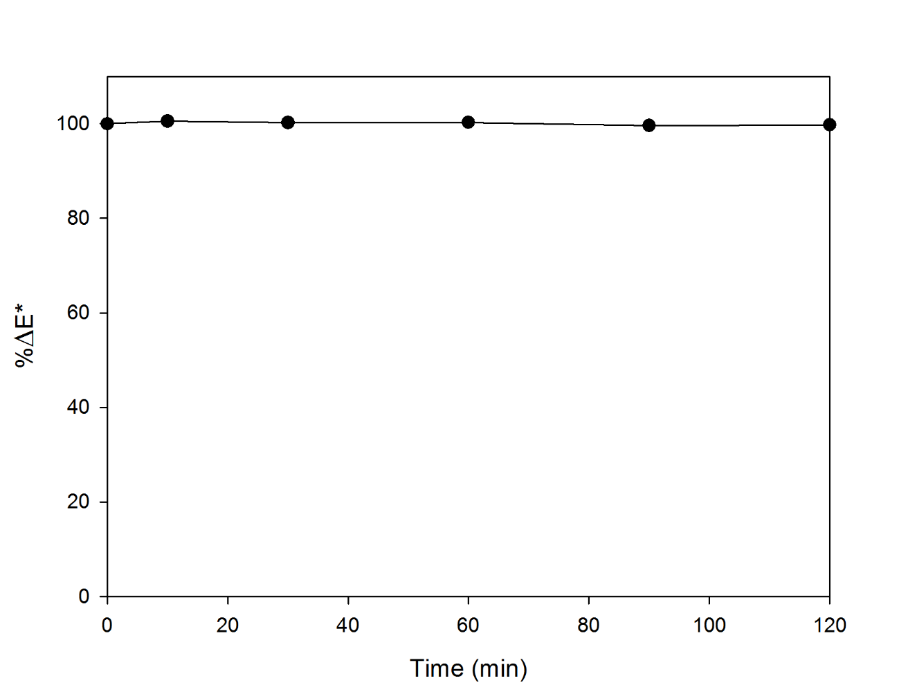


**Figure S8.** Evolution of %ΔE* for a ST12Au treated stone sample stained with methylene blue maintained in dark conditions.


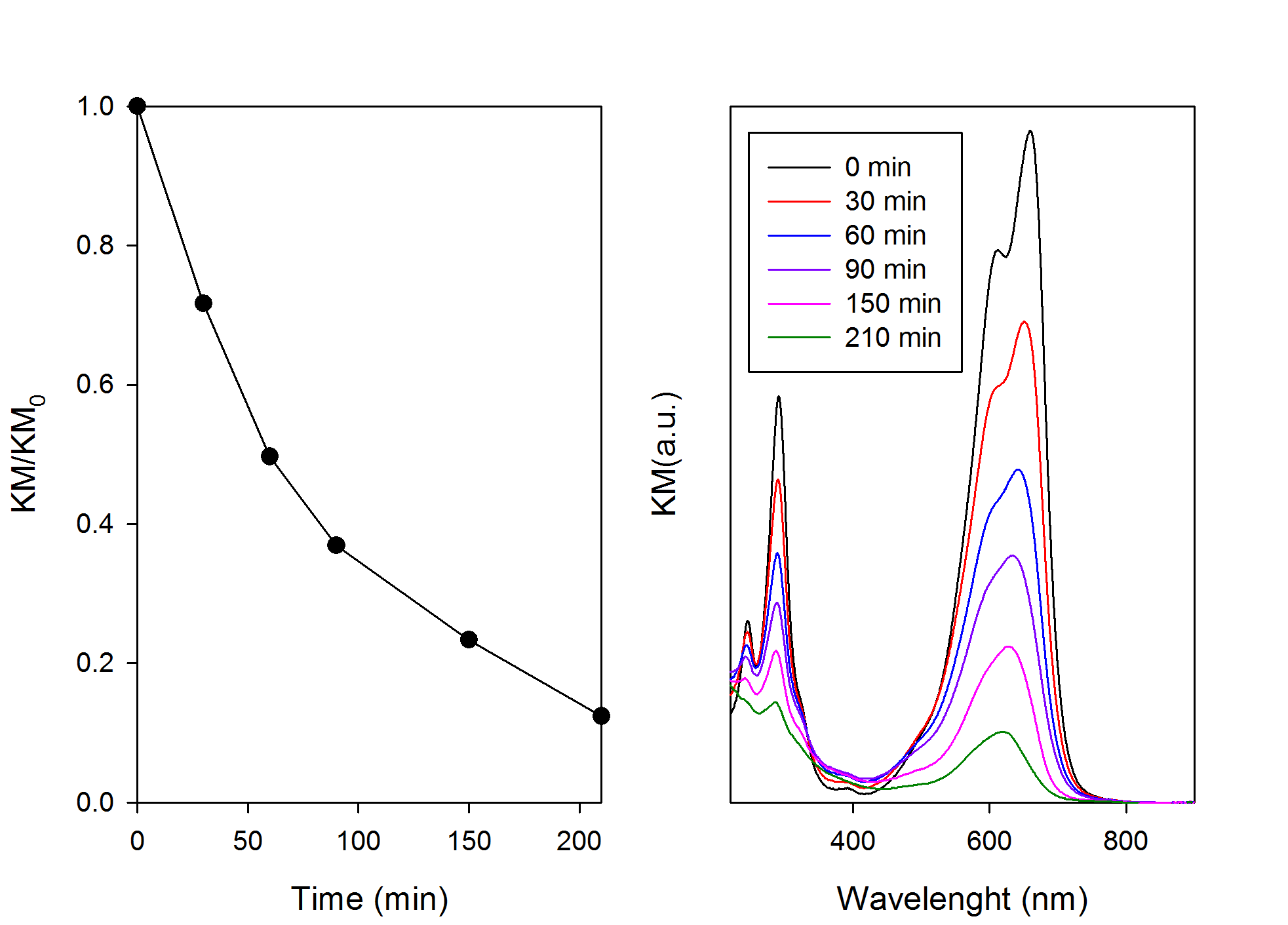


**Figure S9.** Degradation plot and absorbance evolution for MB with the irradiation time.
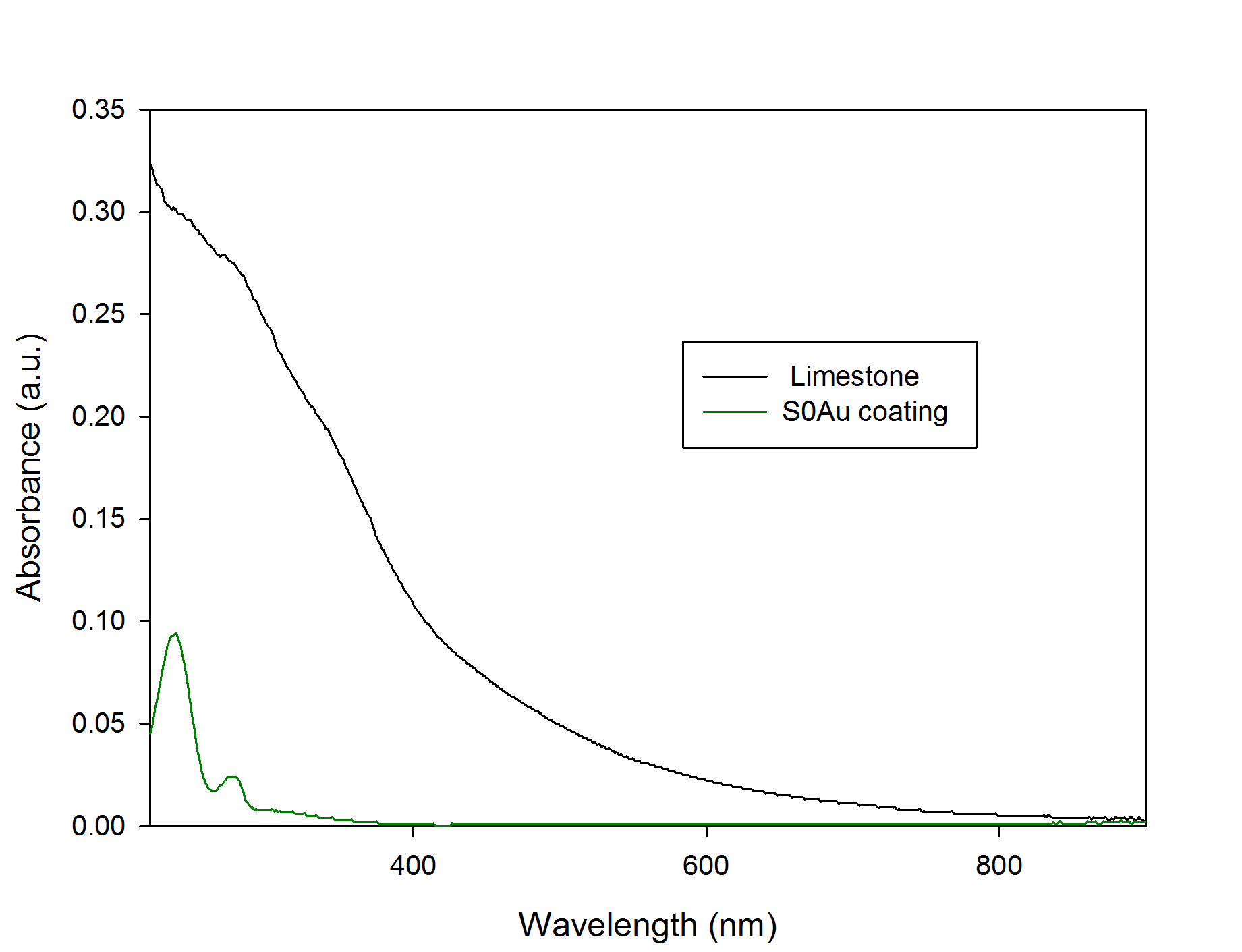
.

**Figure S10.** UV-visible absorbance spectra of S0Au coating and the limestone employed in this work. The absorbance spectra were obtained from respective reflectance spectra.


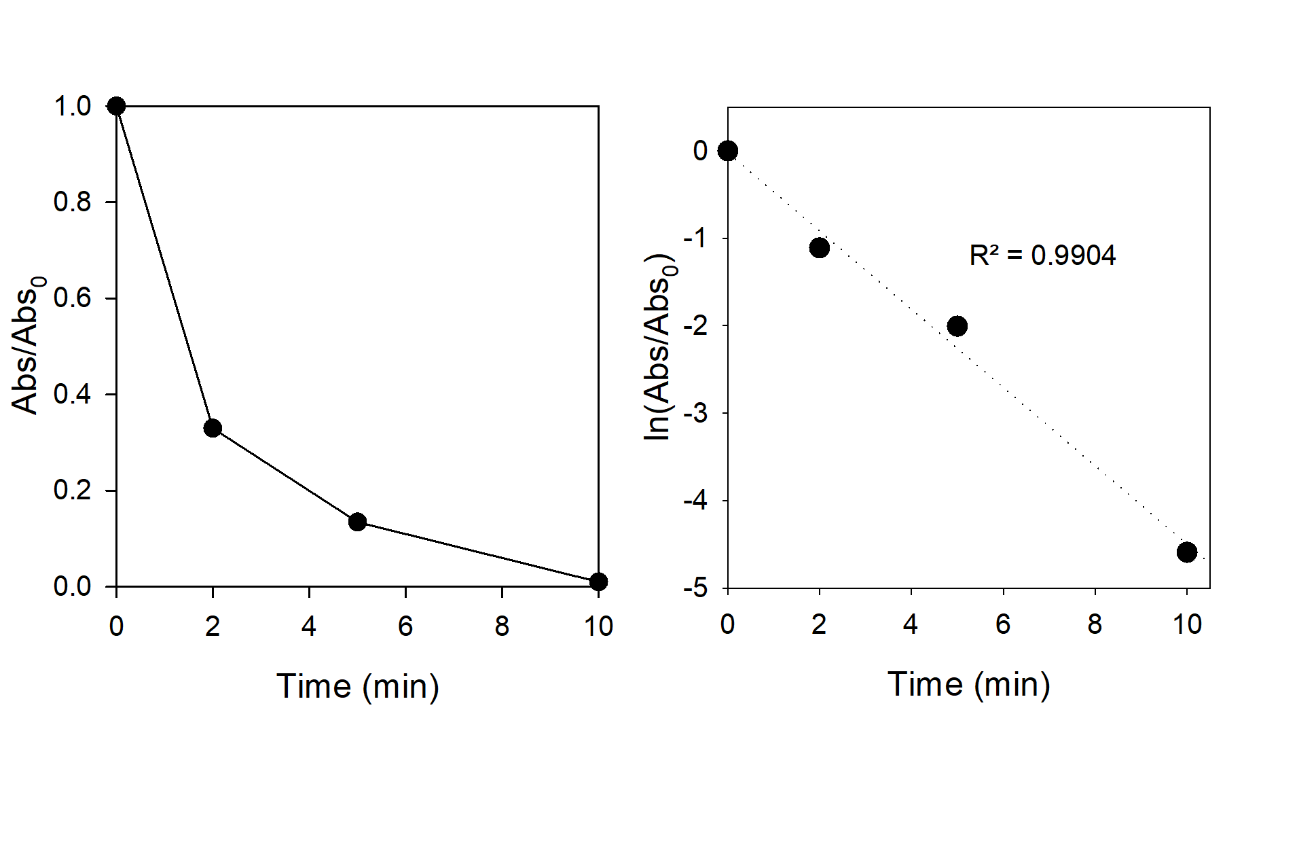


**Figure S11.** Left, evolution of MB degradation on ST0Au coating deposited on glass. Right, its corresponding fitting to first order rate equation.


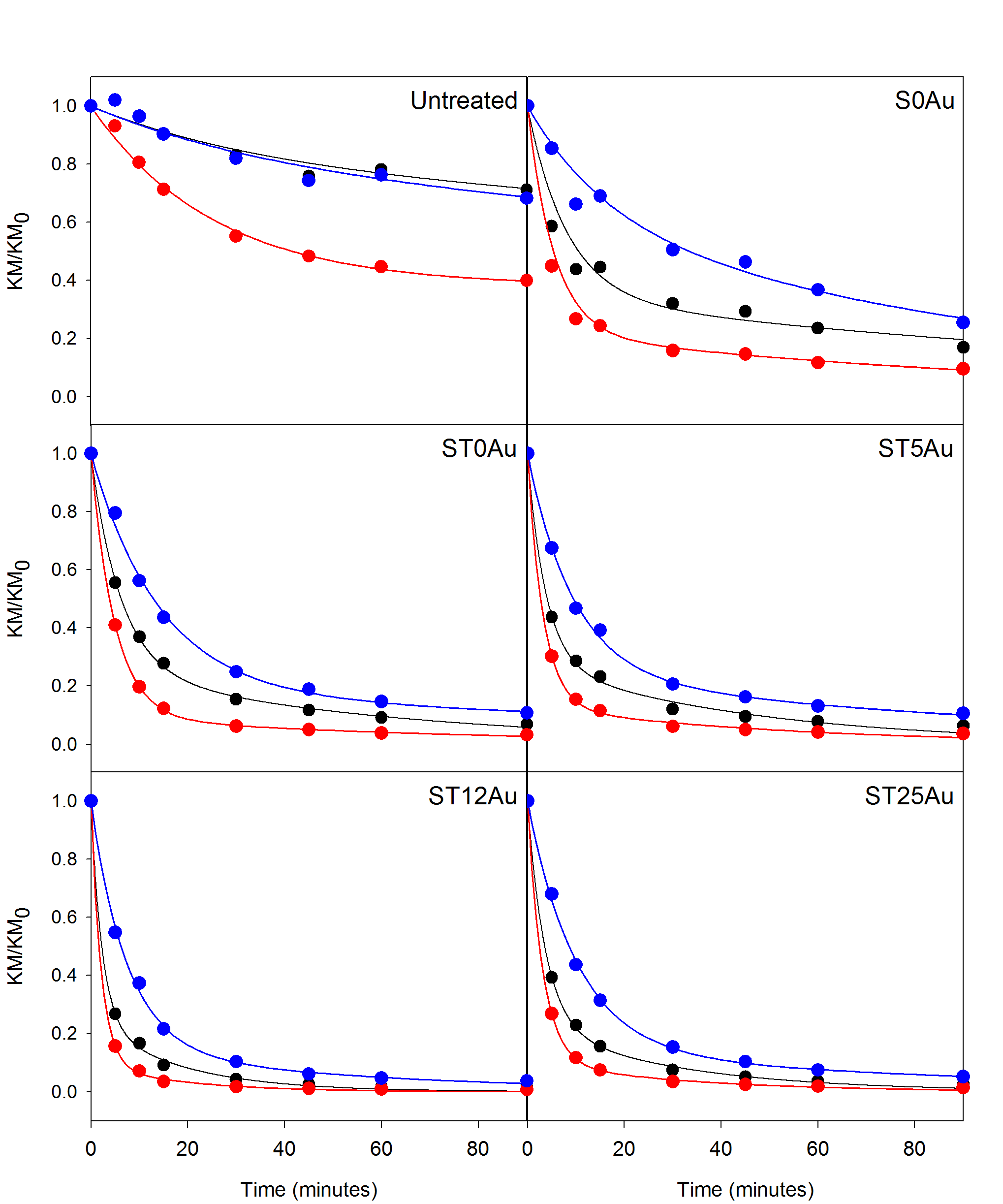


**Figure S12.** Fitting between the KM/KM_0_ values measured (dots) and calculated by fitting to rate equation (lines) for the different degradations processes, overall (black), monomer (red) and dimer (blue).


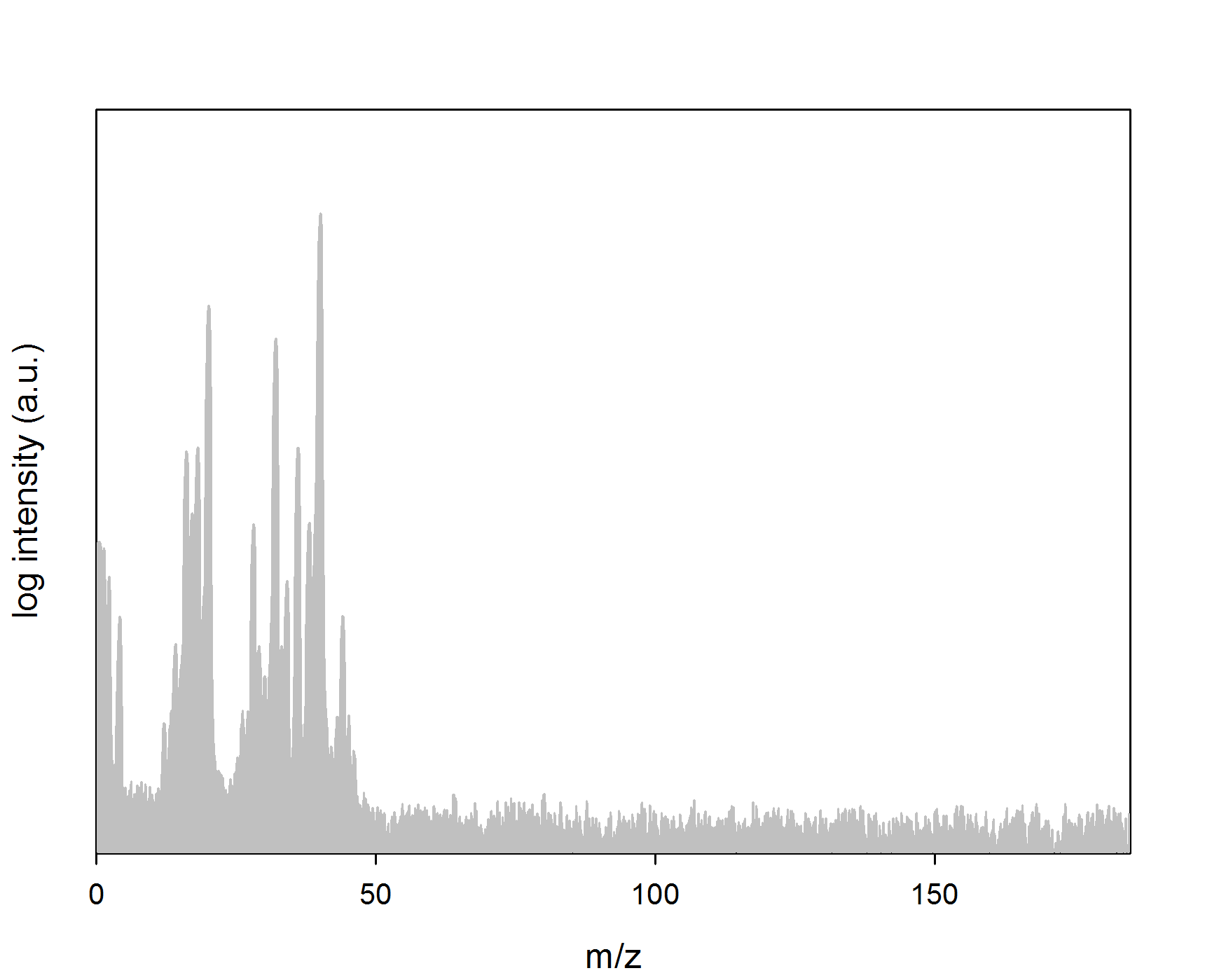


**Figure S13.** Mass spectrum of air from reactor obtained during the mass spectroscopy experiments when the sample was being irradiated with light.
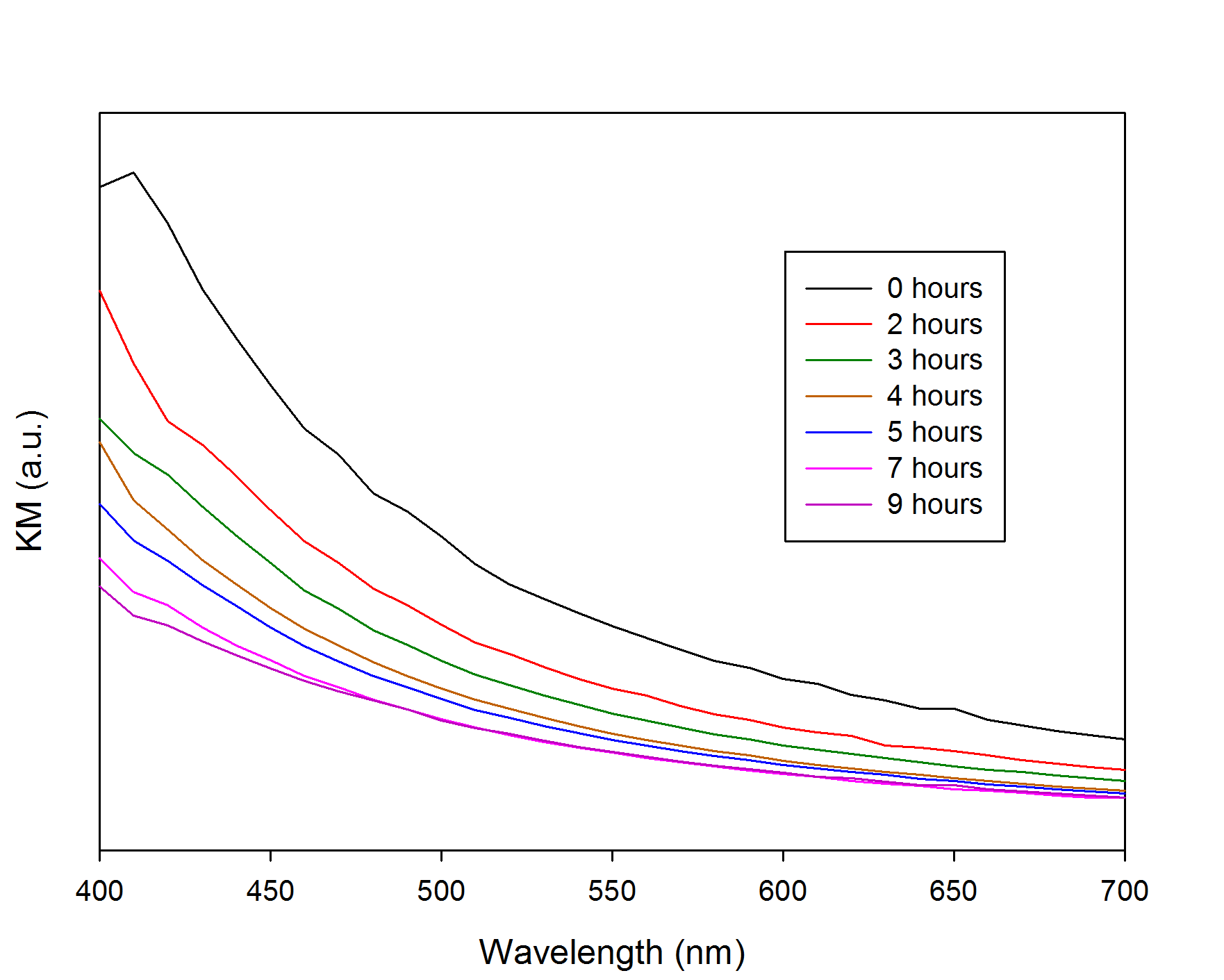
.

**Figure S14.** Example of soot absorbance spectra during soot self-cleaning test for ST12Au treated stone.
